# Distinct Internalization Pathways of Human Amylin Monomers and Its Cytotoxic Oligomers in Pancreatic Cells

**DOI:** 10.1371/journal.pone.0073080

**Published:** 2013-09-03

**Authors:** Saurabh Trikha, Aleksandar M. Jeremic

**Affiliations:** Department of Biological Sciences, The George Washington University, Washington, District of Columbia, United States of America; University of Akron, United States of America

## Abstract

Toxic human amylin oligomers and aggregates are implicated in the pathogenesis of type 2 diabetes mellitus (TTDM). Although recent studies have shown that pancreatic cells can recycle amylin monomers and toxic oligomers, the exact uptake mechanism and trafficking routes of these molecular forms and their significance for amylin toxicity are yet to be determined. Using pancreatic rat insulinoma (RIN-m5F) beta (β)-cells and human islets as model systems we show that monomers and oligomers cross the plasma membrane (PM) through both endocytotic and non-endocytotic (translocation) mechanisms, the predominance of which is dependent on amylin concentrations and incubation times. At low (≤100 nM) concentrations, internalization of amylin monomers in pancreatic cells is completely blocked by the selective amylin-receptor (AM-R) antagonist, AC-187, indicating an AM-R dependent mechanism. In contrast at cytotoxic (µM) concentrations monomers initially (1 hour) enter pancreatic cells by two distinct mechanisms: translocation and macropinocytosis. However, during the late stage (24 hours) monomers internalize by a clathrin-dependent but AM-R and macropinocytotic independent pathway. Like monomers a small fraction of the oligomers initially enter cells by a non-endocytotic mechanism. In contrast a majority of the oligomers at both early (1 hour) and late times (24 hours) traffic with a fluid-phase marker, dextran, to the same endocytotic compartments, the uptake of which is blocked by potent macropinocytotic inhibitors. This led to a significant increase in extra-cellular PM accumulation, in turn potentiating amylin toxicity in pancreatic cells. Our studies suggest that macropinocytosis is a major but not the only clearance mechanism for both amylin’s molecular forms, thereby serving a cyto-protective role in these cells.

## Introduction

Human islet amyloid polypeptide or amylin is a 37- amino acid peptide hormone produced and co-secreted with insulin by pancreatic beta-cells [1]–[4]. Under physiological conditions human amylin in its monomeric state regulates a broad range of biological functions including inhibition of insulin release [5]–[11] and slowing of gastric emptying [5], [12], thereby maintaining glucose homeostasis. Amylin receptor (AM-R) belongs to class-B of the G-protein coupled receptor (GPCR) family. AM-R is formed by physical coupling of another GPCR, a calcitonin receptor (CT-R) with one of the three associated receptor activity modifying proteins (RAMP_1–3_), conferring the receptor’s distinct physiological and pharmacological characteristics [13]–[20]. The biological roles of AM-R however remain obscure.

Under the pathological conditions associated with type 2 diabetes mellitus (TTDM), amylin undergoes a conformational transition from soluble random coil monomers to α-helical oligomers and insoluble β-sheet amyloid fibrils or aggregates [2], [4], [21]–[24]. Reports from several laboratories suggest that oligomers rather than mature fibrils are the main cytotoxic species [25]–[27]. However, other studies show that amylin aggregation may also contribute to the pathogenesis of TTDM [28]–[32]. Both intracellular and extracellular accumulations of human amylin oligomers and aggregates in the pancreas are reported to be cytotoxic to islet β-cells, the loss of which correlates with progression of TTDM [26]–[28], [32], [33]. Direct contact of these oligomers and aggregates with the β-cell PM is required to elicit apoptosis [11], [30], [34]. Amylin-evoked membrane destabilization and cation channel formation in cell membranes are proposed as the two main cytotoxic mechanisms [21], [35]–[38]. Endoplasmic reticulum stress response [39], activations of stress-activated kinases [40], induction of reactive oxidative stress species or radicals [41], [42] and Ca^2+^ overload [35], [43] are other possible mechanisms of amylin-induced toxicity in cells.

Although these studies collectively provide a link between amylin aggregation and its toxicity, currently little is known about the mechanisms that mediate turnover and clearance of amylin’s monomeric and cytotoxic oligomeric forms in cells. One possibility is that amylin is taken in by clathrin mediated endocytosis (CME). This canonical endocytotic pathway is initiated by ligand binding to the specific receptor on the PM, which in turn simulates formation of clathrin coated pits and internalization of the receptor-ligand complex [44]–[48]. CME require GTPase dynamin as an adaptor protein in pinching of clathrin coated vesicles from the cell surface [45]. The clathrin coat then depolymerizes followed by delivery of the cargo to early endosomes and then to late endosomes/lysosomes for degradation or to recycling endosomes for recycling of the receptor back to the PM [45]. Although the evidence for human amylin internalization pathways, particularly in pancreatic cells is scarce [4], [49], [50], many studies have revealed the involvement of a multitude of endocytotic pathways in uptake of other amyloid peptides in a variety of cell types and tissues [47], [51]–[54]. For example, low density lipoprotein 1 receptor has been shown to internalize soluble forms of β-amyloid complexed with apolipoprotein E mainly through the CME in neuroblastoma and neuronal cell lines [52]. Also, *α*7 nicotinic cholinergic receptor transfected human neuroblastomas decrease soluble β-amyloid uptake after treatment with phenylarsine oxide, an inhibitor of clathrin-coat formation, and further indicating involvement of clathrin in this uptake process [52]. In addition to CME, amylin may also be taken in by clathrin independent endocytosis (CIE). Two distinct dynamin-independent and- dependent CIE pathways have been reported [45], [48], [54], [55]. Several GTPases (CDC42, Rho-A or ARF-6) have also been reported to be involved in different CIE pathways and modifying the function of any of these GTPases can affect the internalization of one set of CIE markers but not others [45], [48], [54], [55]. The dynamin-dependent CIE pathway is dependent on RhoA GTPase and caveolae, which are flask shaped invaginations on the PM characterized by caveolin protein [45], [54], [55]. On the other hand, the dynamin-independent CIE pathway depends on small GTPases, CDC42 or ARF6 [45], [54], [55]. CIE of β-amyloid has been reported in several neuronal cell lines types [52]. Soluble forms of oligomeric β-amyloid undergo dynamin-mediated and Rho-A-regulated endocytosis in cultured Neuro-2A cells [51], [54]. Also, α-synuclein uptake has been shown to be markedly reduced by suppression of dynamin [51]. In cervical sympathetic neurons, β-amyloid oligomer uptake occurs at lipid rafts, possibly via monosialotetrahexosylganglioside (GM-1) as reductions of cellular cholesterol and sphingolipid levels in the lipid rafts significantly attenuate uptake [52]. A third process that may bring amylin into the cells is a type of receptor independent endocytosis, macropinocytosis, responsible for the uptake of solutes from the extra-cellular solution [45]–[48], [56]. It is characterized by actin and tubulin polymerization, which are required for proper closure of membrane ruffles and formation of macropinocytotic vesicles ranging in size from 0.5–5 µm, and a non-saturable uptake mechanism [45]–[48], [56]. The soluble oligomeric forms of β-amyloid have been shown to internalize in microglia cells via fluid-phase macropinocytosis with the uptake of fibrillar forms of β-amyloid predominantly via phagocytosis [47]. Apart from endocytosis, direct penetration (translocation) has also been suggested as another uptake mechanism for soluble forms of β-amyloid, primarily in neurons but not other cell types [52], [57], [58], the entry of which is insensitive to low temperatures, known to block all endocytotic mechanisms [50], [57], [58]. Consistent with this fact, human amylin was proposed to translocate the PM by a non-endocytotic mechanism, commonly used by antimicrobial and other cationic cell penetrating peptides (CPPs) [50], [58], [59].

By analogy to the diverse internalization pathways, described for other amyloid proteins, we hypothesized that one or more of the above mentioned uptake mechanisms may regulate human amylin monomer and/or oligomer turnover and toxicity in pancreatic cells. We found that at non-toxic (nM) concentrations monomers internalize in pancreatic cells by an AM-R dependent mechanism. In contrast, at higher cytotoxic (µM) concentrations which produce oligomers, amylin initially was taken in by macropinocytosis and to a lesser extent by translocation. Later, following partial clearance, the remaining extracellular monomers internalize through a clathrin dependent but AM-R- and macropinocytotic-independent pathway. On the other hand, amylin oligomers at this late stage utilize macropinocytosis as primary mechanism to enter these cells, the inhibition of which stimulate their extracellular/PM accumulation and toxicity. Thus, several distinct internalization pathways, in particular receptor-independent macropinocytosis, regulate uptake and clearance of amylin monomers and its cytotoxic oligomers in pancreatic cells in a concentration and time dependent manner.

## Materials and Methods

### Reagents

5-(N-ethyl-N-isopropyl)-amiloride (EIPA), Cytochalasin-D (CytD) and methylene blue (MB) were purchased from Sigma. Chlorpromazine (Chl) and wortmannin (Wort) were obtained from Calbiochem. Dynasore (Dyn) and AC-187 were purchased from Tocris. Alexa-Fluor labeled Cholera Toxin (CTX), transferrin (Trf) and dextran (10,000 MW) were from Invitrogen. Human amylin, RAMP1, RAMP2 and RAMP3 isotype specific rabbit polyclonal and CTR goat polyclonal primary antibodies were all purchased from Santa Cruz Biotechnology (Dallas, TX). Conformation specific rabbit polyclonal oligomer A11 antibody was purchased from Invitrogen (Grand Island, NY).

### Preparation of Human Amylin

Lyophilized C-terminal amidated synthetic human amylin (American Peptide Co., Sunnyvale, CA) was used for preparation of amylin stock solutions (500 µM) using hexafluoride isopropyl alcohol (HFIP) as a solvent. The appropriate amount of peptide was solubilized in HFIP overnight to completely dissolve amylin. This approach efficiently removes any preformed amylin oligomers and aggregates [4]. Before experiments, HFIP was evaporated under a stream of dry nitrogen gas to remove the organic solvent, which is toxic to cells. Human amylin was then reconstituted using freshly prepared culture medium to final concentrations of either 100 nM or 10 µM.

### Cell Cultures and Treatments

Pancreatic rat insulinoma (RIN-m5F) β-cells (ATCC, Gaithersburg, MD ) were cultured in RPMI 1640 medium (ATCC) supplemented with 10% (v/v) fetal bovine serum and 1% penicillin/streptomycin at 37°C in a humidified incubator with 5% CO_2_. Cells were passaged bi-weekly. Passages 10–50 were used for all the experiments. 150 µl of RIN-m5F cells were plated at density 50,000 cells/well and cultured for 24 hours prior to the experiments with human amylin. Human islets from non-diabetics with >90% purity and viability were obtained through the NIH Pilot Program for Human Islet Research. Upon arrival, islets were further purified by handpicking (≥98% based on dithizone staining), gently dissociated in TrypLe cell dissociation medium (Invitrogen) using a 1ml-glass pipette and plated on 96-well glass bottom SensoPlates (Greiner Bio-One) precoated with poly-D-Lysine for confocal studies. Pancreatic cells were treated with pharmacological inhibitors, EIPA (100 µM), CytD (10 µM), Wort (300 nM), Dyn (80 µM) or Chl (25 µM) for 1 hour followed by addition of human amylin at indicated concentrations for either 1 hour or 24 hours. AC-187 (1–100 nM) or MB (100 µM) was co-incubated with human amylin for total duration of studies (24 hours).

### Plasmids and Cell Transfection

Plasmids encoding wild type (wt) clathrin-adaptor protein, wt-AP180, and its C-terminal FLAG dominant-negative (DN) AP180CFLAG were kindly provided by Julie Donaldson (NIH). A DN dynamin 1 mutant construct, DN-dyn1K44A, was obtained from Addgene. The control (empty) vector construct, pcDNA3.1 was a gift from Prof. Damien O’Halloran (GWU, Dept. of Biology). RIN-m5F cells were initially seeded onto glass SensoPlates in serum containing culture medium for 24 hours. Once they reached 60–70% confluence, the cells were washed and replaced with serum- and antibiotic-free media. 1 µg of plasmid DNA and 2 µl of Fugene HD transfection reagent (Promega) were added to the cells for an additional 16–18 hours to allow for transfection. The transfection medium was replaced with normal culture medium and cells allowed to recover overnight. The RIN-m5F cells were then incubated with either 100 nM or 10 µM of human amylin for additional 24 hours. Staining with anti-FLAG antibody revealed the efficiency of transfections to be ≥85%.

### Immuno-Confocal Microscopy

Following amylin treatment, cultured RIN-m5F or human islet pancreatic cells were further incubated with alexa-fluor labeled endocytotic markers, CTX, Trf or Dextran for 30 minutes at 37°C or 4°C. Cells were washed, fixed with 4% paraformaldehyde and permeabilized. Membrane-bound and internalized monomers and oligomers were detected by incubating the cells with rabbit anti-human amylin antibody (1∶200) or rabbit anti-oligomeric A11 antibody (1∶200) at 4°C overnight, respectively. Cells were then exposed to goat anti-rabbit Alexa 488-conjugated secondary antibody (1∶300) for 60 minutes at room temperature. Three random fields in each well were imaged by confocal microscopy, with each treatment performed in three to nine separate experiments. Single optical sections (1µm *z* axis) through the middle of the cells were acquired for each field using an LSCM-510 Meta confocal microscope (Carl Zeiss, Thornwood, NY) equipped with 63X and 100X (1.4 N.A.) oil objectives. The pinhole was adjusted to keep the same size of *z*-optical sections for all channels and objectives used. Multitrack imaging was performed to ensure that there was no crosstalk between the channels. The intensity thresholds of the channels were set using the Zeiss cross-hair function to avoid an arbitrary background threshold being set. In that way, the background pixels that might contribute to false co-localization were excluded from analysis. The Pearson co-localization coefficient (*R*) was determined using Zeiss co-localization software (release 4.0). Images were assembled using Adobe Photoshop software (Adobe Systems Co.) The extent of amylin internalization and accumulation on the PM of pancreatic cells was determined using the Zeiss histogram analysis function (release 4.0) as explained previously [4]. The percentages of amylin are expressed relative to the total amylin associated with the cells (taken as 100%) not considering the amylin in the medium. The relative and absolute changes in amylin extracellular concentrations were analyzed by western blot and ELISA, respectively (explained below).

### Time Lapse Analysis of Extracellular Amylin

Amylin monomer and oligomer accumulation in the media was estimated by Western blot. Pancreatic cells were incubated with culturing medium containing or lacking macropinocytotic inhibitors for 1 hour followed by addition of 10 µM freshly prepared human amylin for 24 hours. 50 µl samples of cell culture media were collected at regular time intervals during 24 hours and proteins in the samples separated by 4–20% Tris-Glycine SDS-PAGE for detection of oligomers or 4–15% Tris-Tricine SDS-PAGE to detect monomers. The proteins were blotted onto nitrocellulose membranes for detection with A11 anti-oligomer antibody (Invitrogen) or human amylin antibody (Santa Cruz) at 1∶2000 and 1∶500 dilutions respectively for 1 hour. This was followed by addition of horseradish peroxidase-conjugated goat anti-rabbit secondary antibody at 1∶5000 dilutions (Thermo Fisher). Blots were developed using ECL substrate (Pierce) and documented using the Kodak 1500 imaging station and densitometry software. Data were expressed as relative optical density units (O.D).

Following similar treatments, real time changes in human amylin concentrations in the RIN-m5F β-cells culture media were quantified by a human amylin ELISA kit (Millipore) according to manufacturer’s guidelines. Prior to analysis, samples were collected and diluted 10,000X for their concentrations to be in the linear range, established by running the standards provided in the kit.

### Cell Toxicity Assays

We used 10 µM amylin concentration, commonly adopted across the field, to generate amylin oligomers and aggregates to study amylin toxicity in cells [4], [50], [60], [61]. RIN-m5F and human islet cells were plated onto 96 well plates for 24 hours followed by incubation with various reagents in the absence or presence of human amylin for additional 24 hours. 3-(4,5-dimethylthiazol-2-)-2,5-diphenyl tetrazolium bromide (MTT) reduction assays (Sigma), lactate dehydrogenase (LDH) release assays (Roche), and caspase-3/7 cleavage (Promega) apoptotic assays were used to quantify the toxic effects of human amylin exerted on cells as described previously [4].

### Statistical Analysis

The Graph Pad Prism 5 Program was used for data plotting and statistical analysis. The unpaired Student’s *t* test or one-way ANOVA followed by the Dunnett-Square or Newman-Keul *post-hoc* test were used for pair wise comparisons among groups when appropriate with significance established at *p<*0.05.

## Results

### Amylin Receptor Dependent and Independent Routes of Human Amylin Internalization in Pancreatic Cells

**Figure 1:**
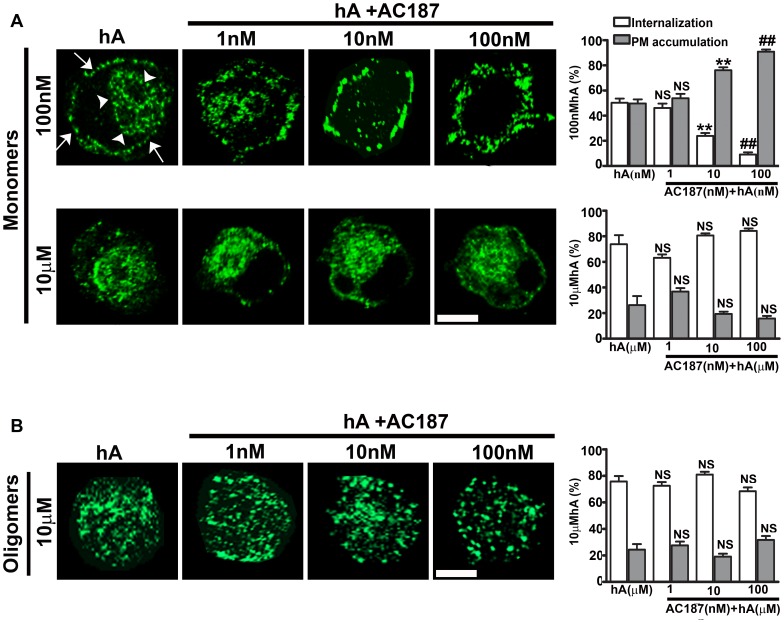
Amylin receptor-dependent and -independent internalization of human amylin in RIN-m5F cells. (**A**) Cells were incubated with 100 nM or 10 µM of human amylin either in the absence or presence of AC-187 (1–100 nM) for 24 hours. Cellular distributions of amylin monomers were detected with human amylin specific antibody. Immuno-confocal microscopy and whole cell analysis revealed that monomer internalization at 100 nM was significantly inhibited with increasing concentrations of AC-187 (top panel, graph). On the contrary, monomer uptake at 10 µM was unchanged upon addition of AC-187 (bottom panel, graph). Arrowheads and arrows denote cells with internalized and PM associated human amylin, respectively. (**B**) Cellular distributions of amylin oligomers, formed at high (10 µM) concentration and detected with A11 antibody were not significantly affected by increasing concentrations of AC-187. **P<0.01, hA 100 nM vs. hA 100 nM/10nM AC187, **^##^**P<0.01, hA 100 nM vs. hA 100 nM/100 nM AC187, NS P>0.1, hA 100 nM vs. hA 100 nM/1 nM AC187 and NS P>0.1, hA 10µM vs. hA 10µM/treatments, n = 9. Significance established by ANOVA followed by Dunnett-Square test. Bar 5µm.

It is well documented that human amylin is toxic to pancreatic rat and human islet cells [4], [50], [60], [61]. However, the exact mechanism and endocytotic machinery regulating amylin turnover in pancreatic cells remain largely unknown. Using immunoconfocal microscopy, we investigated the roles of amylin receptor (AM-R) and endocytosis in the uptake and toxicity of human amylin in cultured pancreatic RIN-m5F and human islet cells. As human amylin is non-toxic at low (nM) concentrations and cytotoxic at higher (µM) concentrations [50], we examined the mechanism of amylin monomer and oligomer internalization at these two distinct concentrations, aiming to understand how cells deal with amylin overload. Hence, cells were incubated for 24 hours with low (100 nM) or high (10 µM) concentrations of freshly prepared human amylin and its intracellular/PM accumulation determined by quantitative immunoconfocal analysis (Figure 1, Figure S1) [4]. Prolonged incubation of cells with 100 nM human amylin allowed amylin accumulation both on the PM and in the perinuclear compartments (Figure 1A, top panel). Whole cell analysis (Figure 1A, top panel and graph) revealed that monomers were equally distributed between PM and intracellular compartments. Incubations of cells with 10 µM human amylin increased intracellular accumulations of monomeric amylin by ∼20% (Figure 1A, bottom panel and graph), indicating a saturable and possibly receptor dependent mechanism for amylin uptake. To confirm or refute a receptor dependent mechanism for amylin monomer uptake, cells were co-incubated with human amylin (100 nM or 10 µM) and the selective amylin receptor antagonist, AC-187 [13], [62]–[65] (1–100 nM) for 24 hours. Immunocytochemistry revealed a dose-dependent inhibition of amylin monomer uptake and its concomitant accumulation on the PM in RIN-m5F cells at low (100 nM) human amylin concentration (Figure 1A, top panel and graph) and in human islets (Figure S1A, top panel and graph) indicating a receptor-dependent mechanism in both cell types. When a high 10 µM amylin concentration was used, the extent of human amylin monomer internalization was not significantly changed by AC-187 in RIN-m5F cells (Figure 1A, bottom panel and graph) or in human islets (Figure S1A, bottom panel, and graph), suggesting an AM-R-independent uptake mechanism. Thus, our results indicate that the mechanism of amylin monomer internalization is dependent on its concentration.

Although, the receptor for amylin has been identified and cloned [13], [14], [18]–[20], its expression in pancreatic cells and its contribution to amylin signaling and toxicity remains enigmatic. Using western blot analysis and isotype specific antibodies, we detected co-expression of RAMP_2_ and CT-R in RIN-m5F cells and RAMP_1_ and CT-R in human islets, reflecting expressions of type-2 and type-1 AM-R respectively (Figure S2A). However, no signal was detected using the RAMP_3_ antibody, indicating no or low expression of the type-3 AM-R isoform in these two cell types (data not shown). In a concentration-dependent manner (1–100 nM), human amylin stimulated expression of type-2 AM-R in RIN-m5F cells as evident by increased co-trafficking of AM-R constituents, RAMP_2_ and CT-R to the PM (Figure S2B, top panel). Similarly, upon addition of human amylin (1–100 nM), there was an increased expression of type-1 AM-R on the PM of human islets (Figure S2B, bottom panel), indicating human amylin-evoked AM-R turnover in these cells. Insulin release assay further revealed a functional coupling between human amylin and AM-R in pancreatic cells as supplementation of AC-187 to the culturing medium revoked the inhibitory effect of human amylin on glucose-evoked insulin release in a dose-dependent manner from RIN-m5F cells (data not shown), and from human islets (Figure S2C).

Next, we tested the involvement of the AM-R in internalization of amylin oligomers. In RINm5F beta-cells incubated with freshly prepared human amylin (10 µM) for 24 hours (controls), 76±4% of the oligomers were found intracellularly whereas 24±4% on the PM of (Figure 1B, graph). In human islets, oligomers were equally distributed between PM and cytosolic compartments (Figure S1B, graph). Interestingly in both cell types, AC-187 failed to prevent amylin oligomer internalization (Figure 1B, Figure S1B), indicating that the AM-R is not involved in uptake of these toxic species.

**Figure 2:**
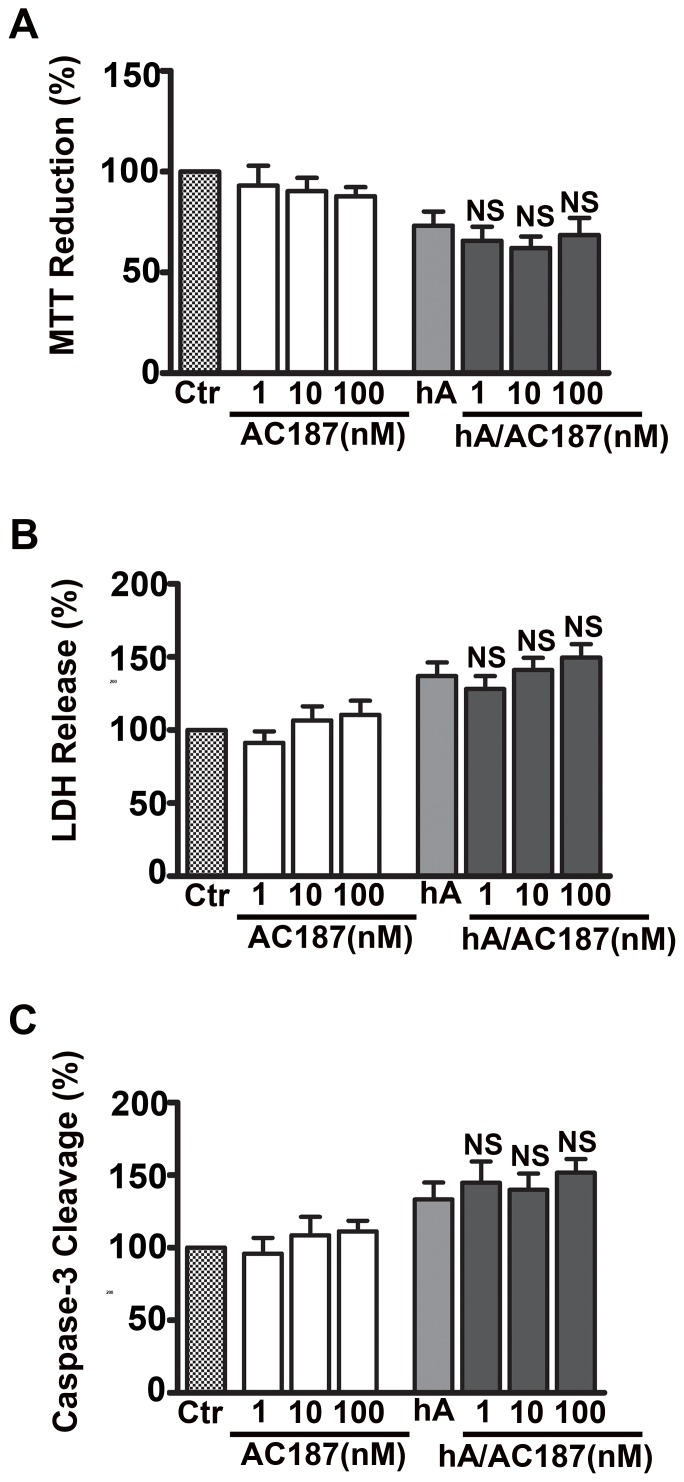
Amylin toxicity is not mediated by amylin receptor in RIN-m5F cells. Cells were treated with 10 µM human amylin for 24 hours either in the absence or presence of AC-187 and the extent of cellular injury determined by MTT reduction (**A**), LDH release (**B**) and Caspase-3/7 cleavage (**C**) assays. AM-R antagonist had no significant effect on human amylin-evoked mitochondrial dysfunction (**A**), LDH membrane leakage (**B**) or activation of pro-apoptotic caspase-3/7 (**C**) as compared to human amylin alone. NS P>0.1, hA vs. hA/treatments, n = 9. Significance established by ANOVA followed by Dunnett-Square test.

After establishing the importance of AM-R in the internalization of amylin monomers (at non-toxic concentrations), we sought to determine the role of AM-R in amylin toxicity. It has been recently shown that AM-R antagonists, AC-187 and AC253 reverse toxicity of human amylin and β-amyloid in human fetal neurons and HEK293 cells, although this required extensive pre-incubation times and equimolar (µM) concentrations of antagonists [62], [63], [65]. In accordance with previous studies [4], [60], [61], human amylin compromised viability of RIN-m5F cells as evident by a significant 23±7% decrease in mitochondrial activity (Figure 2A), a 37±9% increase in LDH release (Figure 2B) and a 33±11% increase in apoptotic caspase-3 activity (Figure 2C) relative to controls (set at 100%). Similar detrimental effect of human amylin on viability was demonstrated in human islets as demonstrated by a 31±4% decrease in mitochondrial activity (Figure S3A), a 36±17% increase in LDH release (Figure S3B) and a 34±10% increase in apoptotic caspase-3 activity (Figure S3C) relative to controls. In contrast to its modulatory effect on amylin-mediated glucose-evoked insulin release from human islets (Figure S2C), AC-187 did not have any significant effect on amylin toxicity in either rat (Figure 2A–C) or human pancreatic cells (Figure S3A–C), indicating an AM-R-independent mechanism of amylin toxicity. Thus, AM-R does not mediate amylin’s toxicity or internalization of its cytotoxic oligomers in either pancreatic cell type.

### Endocytotic and Non-endocytotic Mechanisms Regulate the Early Phase of Human Amylin Internalization

**Figure 3:**
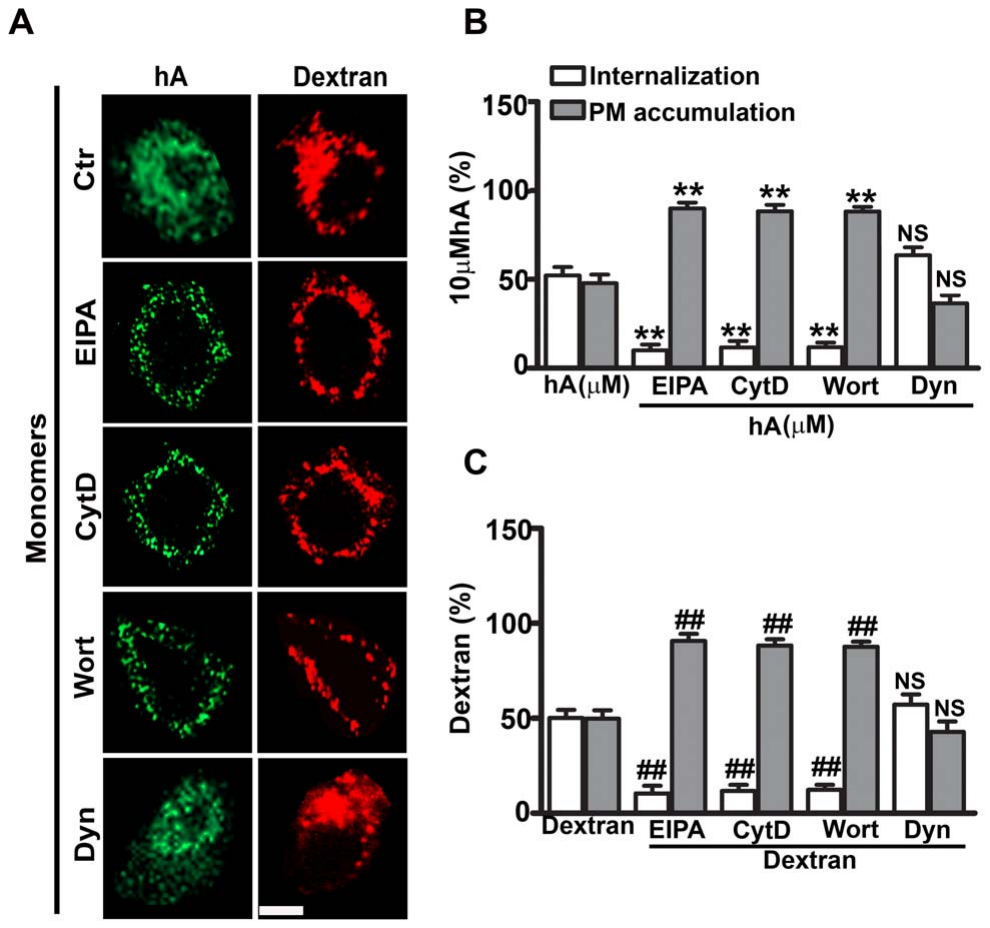
Dynamin-independent macropinocytosis regulates an early stage of amylin monomer internalization in RIN-m5F cells. Cells were treated with EIPA, CytD, Wort or Dyn for 1 hour followed by human amylin (10 µM) incubation for an additional 1 hour at 37°C. Dextran at 40µg/ml was then added for 30 minutes. Confocal microscopy (**A**) and whole cell analysis (**B–C**) revealed reduced internalization and increased PM accumulation of both monomers (**B**) and dextran (**C**) in the presence of the macropinocytotic inhibitors as compared to controls. **P<0.01, hA vs. hA/treatments and ^##^P<0.01, dextran vs. dextran/treatments, n = 9. On the contrary, there was no change in their cellular distributions when treated with Dyn. NS P>0.1, hA vs. hA/Dyn and NS P>0.1, dextran vs. Dextran/Dyn, n = 9. Significance established by ANOVA followed by Dunnett-Square test. Bar 5µm.

**Figure 4:**
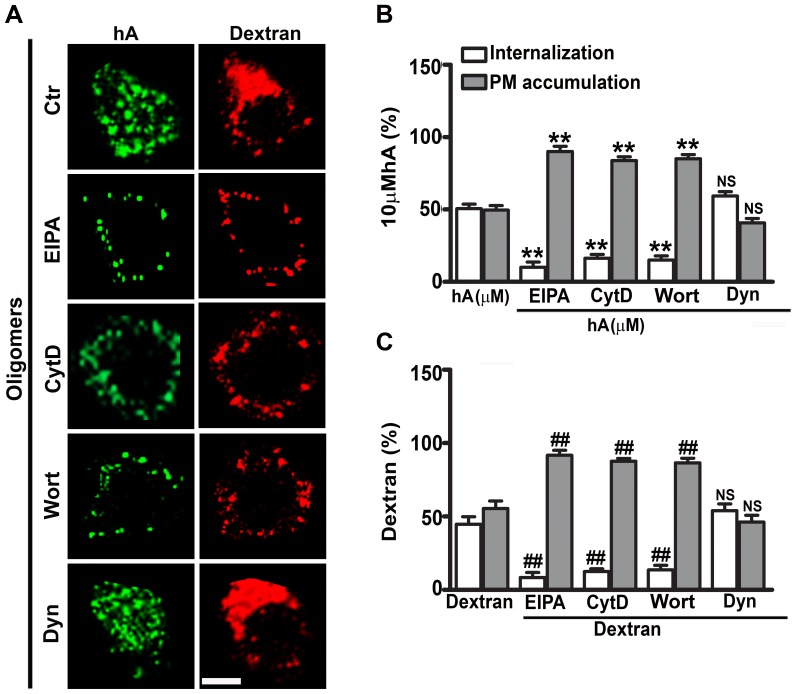
Initial entry of amylin oligomers in RIN-m5F cells is through dynamin-independent macropinocytosis. Cells were treated with EIPA, CytD, Wort or Dyn for 1 hour followed by human amylin (10 µM) incubation for an additional 1 hour at 37°C. Dextran was finally added for 30 minutes. Confocal microscopy (**A**) and whole cell analysis (**B–C**) demonstrated a significant decrease in internalization and an increase in PM accumulation of oligomers (**B**) and dextran (**C**) in the presence macropinocytotic inhibitors when compared to controls. **P<0.01, hA vs. hA/treatments and ^##^P<0.01, dextran vs. dextran/treatments, n = 9. However, Dyn failed to abrogate internalization of oligomers and dextran. NS P>0.1, hA vs. hA/Dyn and NS P>0.1, dextran vs. Dextran/Dyn, n = 9. Significance established by ANOVA followed by Dunnett-Square test. Bar 5µm.

To further dissect the molecular mechanism of amylin monomer and oligomer internalization, confocal microscopy along with specific fluorescent endocytotic markers and pharmacological inhibitors were used. We first examined the mechanism that operates during an early phase (1 hour) of human amylin internalization (Figure 3–4, Figure S4–6). It was previously shown that the small and soluble oligomeric forms of brain derived β-amyloid peptide were avidly taken up by microglia cells through fluid phase-macropinocyotsis [47] which may also play a role in the initial uptake of human amylin in RIN-m5F cells. To test this hypothesis, cells were pre-treated with macropinocytotic inhibitors, EIPA, CytD or Wort, and amylin uptake examined. Under control conditions (no inhibitors), a sizable fraction (52±5%) of amylin monomers internalized in these cells (Figure 3), a portion of which trafficked to dextran-positive intracellular compartments in RIN-m5F cells as evident by their partial co-localization (R = 0.48±0.02, Figure S4A top panel, Figure S4B). This suggests a common macropinocytotic dependent internalization mechanism for dextran and amylin monomers. To further confirm that macropinocytosis is involved in the uptake of amylin monomers, macropinocytotic inhibitors were used (Figure 3). Whole cell analyses revealed that 52±5% of cell-associated amylin monomers (Figure 3A–B) and 50±4% dextran (Figure 3A, C) accumulated in these cells under control conditions, the rest being associated with PM. Inhibition of macropinocytosis by EIPA [45], [46], [48], markedly decreased the internalization of monomers to 10±3% and dextran to 10±4%, and in turn increased their PM accumulation to ≥90% (Figure 3A–C). Consequently, a significant decrease in intracellular co-localization between amylin monomers and dextran was observed (R = 0.07±0.01, Figure S4A top panel, Figure S4B). Macropinocytosis is known to be dependent on actin polymerization. The latter is required for PM ruffling and subsequent formation of macropinosomes [45]–[47].Consistent with fluid-phase uptake mechanism, an inhibitor of actin polymerization, cytD [4], [45]–[47] inhibited internalization of both monomers and dextran by ∼40% causing a comparable increase in their PM accumulation (Figure 3A–C). This treatment also prevented their intracellular co-localization as evident by a very low co-localization (R = 0.07±0.02, Figure S4A top panel, Figure S4B). Other than actin, phosphatidylinositide-3-kinase (PI 3-kinase) is required for macropinocytosis by directing the proper closure of membrane ruffles that leads to the formation of macropinosomes [48], [56], [66], [67]. Hence, cells were pre-incubated with a specific inhibitor of PI 3-kinase, Wort [48], [56], [66], [67] and its effect on amylin internalization examined. As with other macropinocytotic inhibitors, Wort significantly reduced internalization of both monomers and dextran, thereby stimulating their PM accumulation (Figure 3A–C). It has been widely debated whether dynamin is required for macropinocytosis. Both dynamin dependent and independent macropinocytosis were found to operate in cells [46]. To investigate the possible involvement of dynamin in amylin internalization, we used a general dynamin inhibitor, Dyn [46], [48]. Confocal microscopy revealed that Dyn failed to abrogate the internalization of both monomers and dextran (Figure 3A–C, Figure S5A–B) in RIN-m5F cells. However, Dyn effectively blocked internalization of Cholera Toxin (CTX) and Transferrin (Trf) (Figure S5–6), known endocytotic markers of dynamin-dependent pathways [45], [46], [48], [52], [54]. Taken together, our results (Figure 3, Figure S4–5) suggest that amylin monomers, when added at higher (10 µM) concentration, initially internalized in RIN-m5F cells by a dynamin-independent fluid phase-macropinocytotic pathway.

To determine if amylin oligomers follow the same internalization route as monomers, we studied trafficking of oligomers and dextran in RIN-m5F cells with immuno-confocal microscopy. Under control conditions, A11 positive oligomers partially co-localized with dextran-positive intracellular compartments (R = 0.45±0.03, Figure S4A bottom panel, Figure S4C), indicating a common internalization mechanism (macropinocytosis) for these two cargos. Whole cell analyses revealed that 50±3% of amylin oligomers (Figure 4A–B) and 45±5% of dextran (Figure 4A, C) from the total cellular pool accumulated inside these cells. Like monomers (Figure 3), oligomer internalization was diminished to 10±3% with EIPA, 16±3% with cytD and 15±3% with Wort, which in turn increased their accumulation (∼85–90%) on the PM of the RIN-m5F cells (Figure 4A–B). Dextran internalization was also significantly reduced to 8–13% by the same inhibitors (Figure 4A, C). Consequently, all three macropinocytotic inhibitors decreased intracellular co-localization between oligomers and dextran (R≤0.1, Figure S4A bottom panel, Figure S4C), once again suggesting a common internalization mechanism. As shown for monomers, internalization of amylin oligomers and dextran was unchanged by Dyn, indicating a dynamin-independent uptake mechanism for these two cargos (Figure 4A–C, Figure S4A–C, Figure S6A–B).

We previously reported that clathrin is implicated in the later stage (24 hours) of amylin monomer internalization in pancreatic cells [4]. To determine if it is also required for initial entry of monomers, RIN-m5F cells were first pre-treated with a specific clathrin inhibitor, chlorpromazine [4], [54], [68], followed by addition of human amylin for 1 hour. CTX and Trf were then added. Trf but not CTX follows clathrin mediated pathway [45], [46], [48], [52], [54], [57]. Chlorpromazine reduced Trf internalization but had no significant effect on internalization of either amylin monomers (Figure S5A–B) or oligomers (Figure S6A–B) during the first hour. Interestingly, small fractions of amylin monomers (12±2%, Figure S5A–B) and oligomers (13±3%, Figure S6A–B) were internalized even when the cells were incubated at low temperatures (≤4°C), while both CTX and Trf internalization were almost completely blocked (≥92%) at ≤4°C (Figure S5–6). The confocal microscopy also revealed a ∼5–6 fold decrease in the number of cells with internalized amylin monomers (from 62±5% to 12±3%) and oligomers (from 60±6% to 10±2%) at ≤4°C. Therefore, our results demonstrate that amylin monomers and oligomers initially internalize in pancreatic cells by clathrin- and dynamin-independent fluid phase macropinocytosis and to a lesser extent (10–15%) by a non-endocytotic (translocation) mechanism.

### The Later Phase of Human Amylin Monomer Internalization Requires Clathrin but not Dynamin

**Figure 5:**
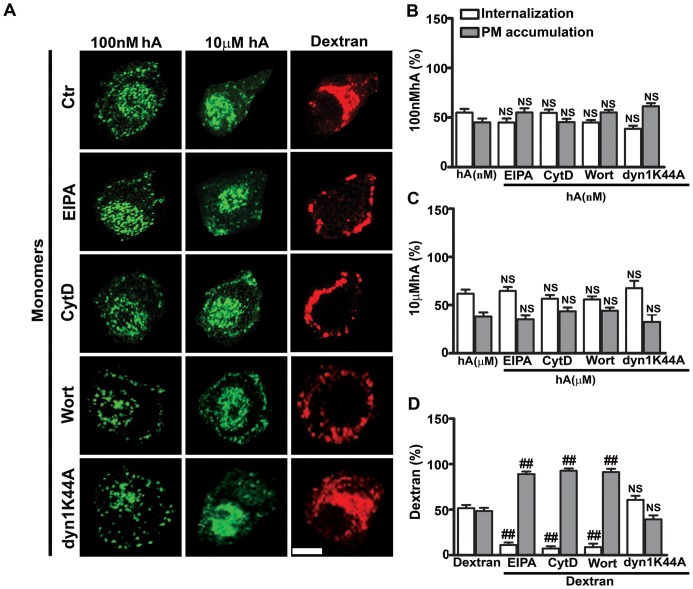
Late phase of amylin monomer internalization in RIN-m5F cells is independent of macropinocytosis. Cells were either treated with EIPA, CytD and Wort for 1 hour or transfected with the dynamin mutant construct, DN dyn1K44A for 16–18 hours followed by human amylin incubation at either 100 nM or 10 µM for an additional 24 hours at 37°C. Dextran was sequentially added after incubating cells with 10 µM human amylin. Confocal microscopy (**A**) and whole cell analysis (**B–D**) showed no change in cellular distributions of monomers at 100 nM (B) or 10 µM (**C**) in the presence of EIPA, CytD, and Wort or DN dyn1K44A when compared to controls. NS P>0.1, hA vs. hA/treatments, n = 9. However, dextran (**D**) internalization was significantly reduced when treated with macropinocytotic inhibitors but not with DN dyn1K44A. ^##^P<0.01, dextran vs. dextran/inhibitors and NS, P>0.1, dextran vs. dextran/dyn1K44A, n = 9. Significance established by ANOVA followed by Dunnett-Square test. Bar 5µm.

We further investigated if macropinocytosis also plays a role in amylin internalization at later times (24 hours). The cells were pre-incubated with EIPA, CytD or Wort for 1 hour and then incubated with low (100 nM) or high (10 µM) amylin for additional 24 hours (Figure 5). This procedure minimizes the toxic effects of these inhibitors, which may interfere with amylin uptake. Following the treatments, dextran was added to the cells. Under control conditions, 55±4% of the cell-associated amylin monomers accumulated inside the cells when incubated with the 100 nM amylin concentration (Figure 5A, B), whereas 62±5% of monomers internalized when challenged with high (10 µM) amylin concentration (Figure 5A, C). This result indicates a saturable uptake mechanism for human amylin, not a characteristic of fluid phase endocytosis [47]. Furthermore, the macropinocytotic inhibitors had no modulatory effect on amylin monomer internalization or PM accumulation respective to controls at 24 hours (Figure 5A–C), while dextran internalization was significantly inhibited by EIPA, CytD and Wort (Figure 5A, D). A very low co-localization value (R = 0.05±0.01) was obtained between amylin monomers and dextran either in the absence or in the presence of these inhibitors (Figure S7A top panel, Figure S7B). Thus, amylin monomers and dextran follow distinct internalization pathways.

**Figure 6:**
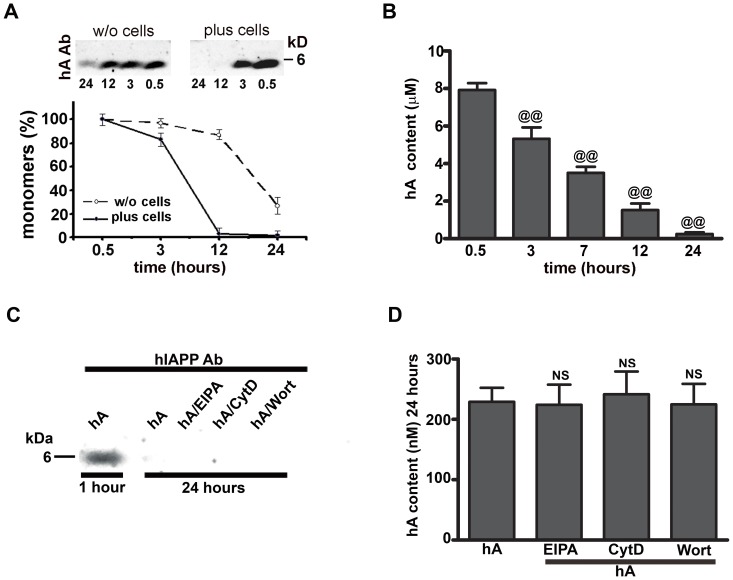
Time lapse analyses of extracellular amylin monomers in RIN-m5F cells. (**A**) Human amylin (10 µM) was added to the cell culture media without (w/o) or containing cells for 24 hours. 50 µl of medium was collected at regular time intervals and amylin resolved on a Tris-Tricine gel. Amylin content was then analyzed by western blot using human amylin antibody (hA Ab). (**B**) Real time changes in extracellular amylin concentrations were analyzed by human amylin-specific ELISA. Upon treatments with macropinocytotic inhibitors (EIPA, CytD or Wort), western blot (**C**) and ELISA (**D**) analyses revealed no significant change in the extracellular human amylin content when compared to only human amylin-treated cells at 24 hours. NS, P>0.1, hA vs. hA/inhibitors, n = 5. Significance established by ANOVA followed by Dunnett-Square test.

The time lapse western blot analyses of extracellular amylin monomers revealed that the majority gradually disappeared from the cell culturing medium within the first 7 hours, with a complete loss of detectable signal at 24 hours (Figure S8). In contrast, amylin monomer levels in cell-free medium did not drop until very late (Figure 6A, left panel, graph). This delayed disappearance of amylin monomers from cell-free medium can be attributed to the peptide’s slow aggregation process described previously [69]. Addition of cells accelerated amylin monomer clearance from the medium, such that at 12 h, ∼5% of the monomers remained in the medium containing cells versus >80% in a cell-free medium (Figure 6A, left panel, graph). In the absence of cells, amylin monomer disappearance could be only due to amylin oligomerization/aggregation and would be independent of cellular uptake/degradation. The marked difference observed in the kinetics of monomer clearance from extracellular medium as compared to cell free medium strongly indicates that endocytosis and/or other cellular process but not aggregation is the major clearance mechanism for extracellular amylin, although peptide polymerization may also be responsible to lesser extent. Using ELISA, we observed that the concentration of human amylin dropped from 8±0.4 µM to 0.24±0.08 µM (Figure 6B) over a period of 24 hours. The macropinocytotic inhibitors had no effect on the clearance of amylin monomers from the culture media measured at 24 hours by western blot (Figure 6C). This finding was further confirmed with ELISA (Figure 6D) showing no significant change in the extra-cellular content of human amylin-treated cells either in the absence or presence of macropinocytotic inhibitors. Therefore, it is likely, that this late phase of amylin internalization is via clathrin and dynamin-dependent endocytotic pathways.

To confirm this, cells were transfected with a full length wild type clathrin construct, wt-AP180 or dominant-negative DN AP180CFLAG mutant construct containing a clathrin binding domain at the C-termini region of AP180, which specifically inhibits clathrin-mediated endocytosis [54], [70], [71]. The cells were sequentially incubated with 100 nM or 10 µM human amylin for 24 hours. Fluorescently tagged CTX and Trf were then added to the cells to label compartments involved in amylin turnover. Expression of wt-AP180 did not significantly change the extent of amylin monomer internalization at 100 nM or 10 µM as compared to its uptake in non-transfected cells (Figure S9A–D). Transfection with the DN AP180CFLAG mutant reduced amylin monomer internalization with a concomitant increase in their PM accumulations at 24 hours at both amylin concentrations (Figure S9A–D). As expected, Trf internalization was significantly reduced in the cells transfected with DN AP180CFLAG (Figure S9C, E, and F). Amylin monomer internalization was blocked at low temperature (≤4°C) (Figure S9A–D) as was uptake of CTX and Trf (Figure S9–10). These observations all support the view that amylin monomers at 24 hours are taken in by clathrin dependent endocytosis.

To probe whether dynamin is involved in amylin monomer internalization at these later times, a plasmid encoding the DN dynamin mutant form (dyn1K44A), deficient in its GTP binding and GTPase activity [54], [70], [72], [73], was used to transfect RIN-m5F cells. Internalization of amylin monomers and dextran were not significantly reduced with respect to the controls in cells expressing the DN dynamin form while CTX and Trf internalization were almost completely blocked indicating a dynamin-independent pathway for human amylin (Figure 5A, D, Figure S7, Figure S11). Taken together, our biochemical and immunoconfocal studies suggest that at later times (24 hours), when their concentration drops to sub-µM range, human amylin monomers change their internalization pathway from dynamin-independent macropinocytosis to clathrin-dependent endocytosis.

### Cytotoxic Amylin Oligomers Internalize by Macropinocytosis in RIN-m5F Cells

**Figure 7:**
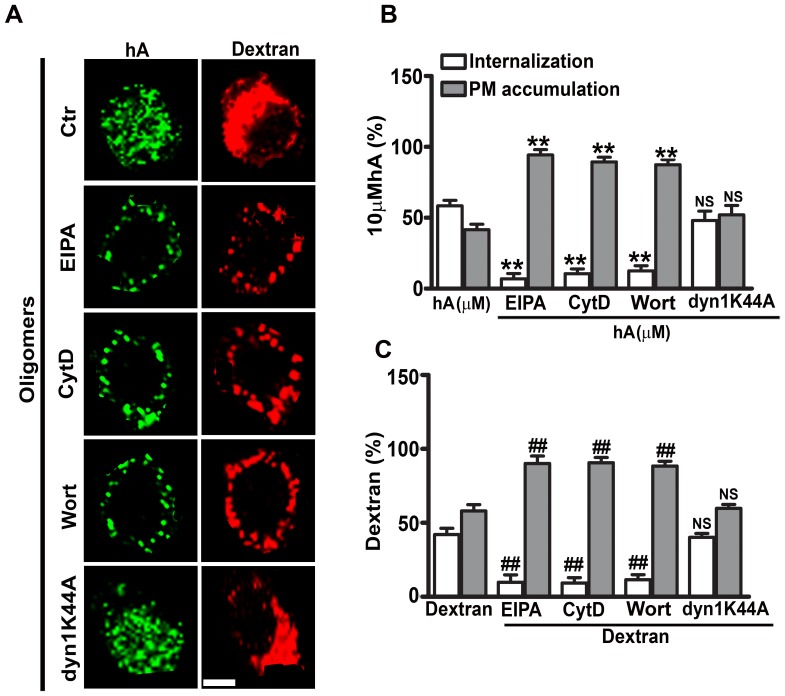
Macropinocytosis is involved in late phase of amylin oligomer internalization in RIN-m5F cells. Cells were either treated with EIPA, CytD and Wort for 1 hour or transfected with dynamin mutant construct, DN dyn1K44A for 16–18 hours followed by human amylin (10 µM) incubation for an additional 24 hours at 37°C. Dextran was finally added for 30 minutes. Confocal microscopy (**A**) and whole cell analysis (**B–C**) demonstrated a significant reduction in internalization and increase in PM accumulation of oligomers (**B**) and dextran (**C**) in the presence of macropinocytotic inhibitors. **P<0.01, hA vs. hA/inhibitors and^ ##^P<0.01, dextran vs. dextran/inhibitors, n = 9. In contrast, expression of DN dyn1K44A in these cells failed to prevent entry of both oligomers and dextran. NS P>0.1, hA vs. hA/dyn1K44A and NS P>0.1, dextran vs. dextran/dyn1K44A, n = 9. Significance established by ANOVA followed by Dunnett-Square test. Bar 5µm.

Following the transfections with DN AP180CFLAG or wild type construct, we observed that the amount of oligomers internalized was similar between controls, wt-AP180- and DN AP180CFLAG-transfected cells (Figure S10A–B), indicating that clathrin was dispensable for oligomer uptake in these cells. Like monomers (Figure S9A–D), oligomer internalization was significantly reduced at low temperatures (≤4°C) (Figure S10A–B), suggesting that amylin oligomers follow endocytosis. A rather minor fraction of oligomers (9±3%) underwent translocation across the PM at this late stage (Figure S10A–B). Internalization of both amylin oligomers (Figure 7A–B, Figure S11A–B) and dextran (Figure 7A, C) was also unaffected by down regulation of dynamin endocytotic function using DN Dyn1K44A. However, the same construct efficiently inhibited CTX and Trf uptakes in RIN-m5F cells (Figure S11A, C and D).

Having established that clathrin and dynamin independent endocytosis account for amylin oligomer internalization at this late stage and taking into account the inhibitory effects of EIPA, CytD or Wort on early (1 hour) oligomer entry, we hypothesized that macropinocytosis is the only endocytotic mechanism of oligomer transport in these cells. Immunofluorescence confocal microscopy revealed that, under control conditions, a relative large pool of A11-positive amylin oligomers co-localize with dextran (R = 0.49±0.03, Figure S7A bottom panel, Figure S7C), indicative of macropinocytosis. To further confirm that this endocytotic pathway accounts for internalization of oligomers, macropinocytotic inhibitors were used (Figure 7). We followed the same internalization protocol as described for monomers (Figure 5). Whole cell analyses of the micrographs revealed that, under control conditions, 59±4% of the cell-associated amylin oligomer pool internalized in RIN-m5F cells, the rest being bound to the PM (Figure 7). Oligomer internalization was markedly reduced in cells treated with EIPA (7±4%), to (10±3%) in CytD treated cells and to (12±4%) with Wort (Figure 7A–B). Likewise, the internalization of dextran was also reduced to (∼10%) using the same inhibitors set (Figure 7A, C), leading to a less intracellular co-localization between oligomers and dextran, (R = 0.1±0.01 Figure S7A bottom panel, Figure S7C), confirming that macropinocytosis regulates uptake of these two cargos in RIN-m5F cells.

**Figure 8:**
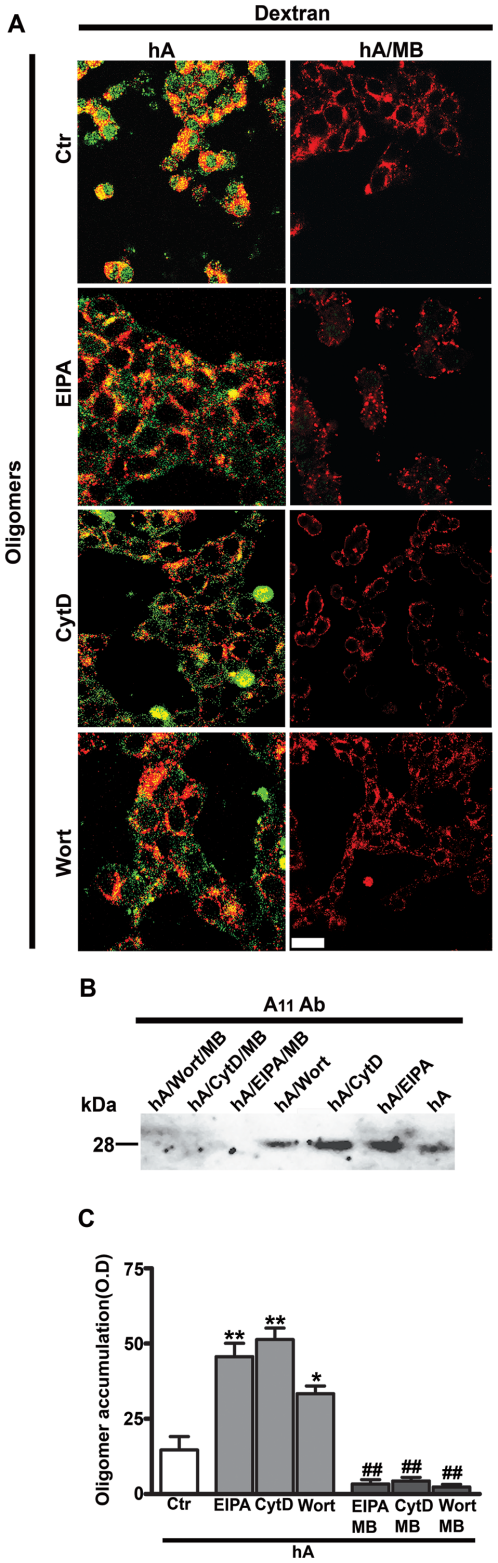
Inhibition of macropinocytosis stimulates amylin oligomerization in RIN-m5F cells. Cells were first treated with macropinocytotic inhibitors (EIPA, CytD or Wort) for 1 hour followed by addition of 10 µM human amylin either in the absence or in the presence of oligomer inhibitor MB (100 µM) for 24 hours. Dextran was added for an additional 30 minutes. (**A**) Immunoconfocal microscopy revealed a significant increase in the PM accumulation of both amylin oligomers (green), detected with A11 antibody, and dextran (red) when treated with these three inhibitors as compared to the controls (human amylin-treated cells) (left panel). However, in the presence of MB, only dextran but not oligomer accumulation was detected on the PM (right panel). Bar 10 µm. (**B–C**) Extracellular amylin oligomers were analyzed by western blot and densitometry. Inhibition of macropinocytosis stimulated a 27–42% increases in extracellular accumulation of intermediate sized ∼25kDa oligomers in the culturing medium as compared to cells treated with human amylin only. This stimulatory effect on oligomer accumulation was reversed by addition of MB. *P<0.05, **P<0.01, hA vs. hA/inhibitors, n = 3, ANOVA followed by Dunnett-Square test and ^##^P<0.01, hA/inhibitors vs. hA/inhibitors/MB, n = 3, ANOVA followed by Newman-Keul post hoc test.

**Figure 9:**
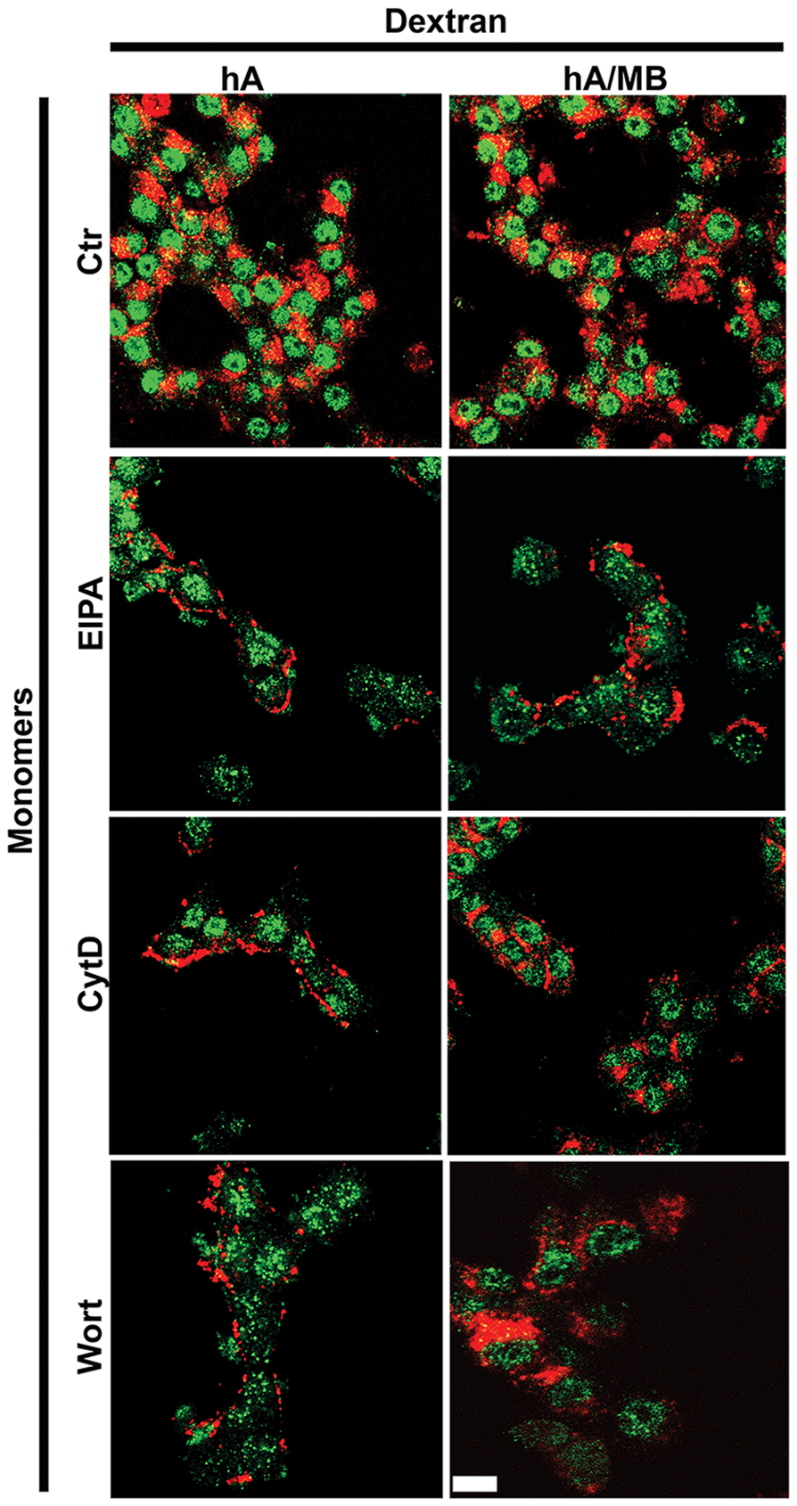
Methylene blue has no inhibitory effect on amylin monomers and dextran in RIN-m5F cells. Cells were first treated with macropinocytotic inhibitors (EIPA, CytD or Wort) for 1 hour followed by addition of human amylin (10 µM) either in the absence or in the presence of MB (100 µM) for 24 hours at 37°C. Dextran was added for an additional 30 minutes. Confocal microscopy revealed no significant change in the cellular distributions of amylin monomers (green) when treated with macropinocytotic inhibitors with or without MB (left and right panel). Also, in the presence of MB, dextran (red) turnover was unaffected (right panel). Bar 10µm.

Studies show that oligomerization of human amylin is a rapid process, which can be further accelerated by its interaction with lipids in the membrane [4], [23]. Given that macropinocytosis regulates the internalization of both molecular forms of amylin during an early phase, it is unclear whether inhibition of monomer or oligomer uptake or both contributes to the extracellular amylin oligomer accumulation at later times (24 hours). It is quite possible that inhibition of amylin monomer internalization could lead to oligomerization and extracellular accumulation. Hence, the stimulatory effect of macropinocytotic inhibitors on amylin oligomer accumulation on the PM of RIN-m5F cells was tested in the absence and presence of the potent and specific amyloid oligomer inhibitor, methylene blue (MB) [4], [74]. Confocal microscopy revealed that MB specifically blocked the formation and deposition of amylin oligomers but not dextran on the PM of these cells (Figure 8A). Similar to dextran, the binding and internalization of amylin monomers were not affected by MB either in the absence or the presence of macropinocytotic inhibitors (Figure 9A). This finding indicates that inhibition of amylin oligomer (Figure 7, 8A) but not monomer (Figure 5, 9A) internalization by these macropinocytotic inhibitors accounts for an increased extracellular oligomer accumulation at later times (24 hours).

In addition to confocal microscopy, the effect of macropinocytotic inhibitors on amylin oligomer turnover and clearance was also examined using a biochemical approach. Western blot analyses revealed the accumulation of intermediate sized oligomers in the culturing medium as compared to human amylin-treated cells (Figure 8B). Densitometry of the corresponding A11 positive bands showed a 27–42% increase in relative amylin oligomer content in the medium of EIPA, CytD or Wort-treated RIN-m5F cells as compared to human amylin-treated cells without inhibitors (Figure 8C). The formation and accumulation of oligomers in the medium were prevented in cells co-incubated with amylin and MB (Figure 8B–C).

### Macropinocytosis Regulates Amylin Oligomer Turnover in Cultured Human Islet Cells

**Figure 10:**
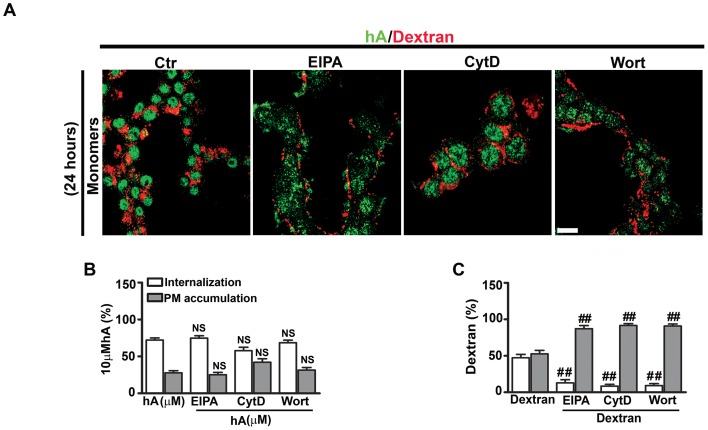
The late phase of amylin monomer internalization does not require macropinocytosis in human islets. Cells were treated with EIPA, CytD or Wort for 1 hour followed by human amylin (10 µM) incubation for an additional 24 hours at 37°C. Dextran was finally added for 30 minutes. Confocal microscopy (**A**) and whole cell analysis (**B–C**) revealed no change in cellular distributions of amylin monomers (green) (**B**), detected with human amylin antibody in the presence of EIPA, CytD or Wort when compared to controls. Dextran (red) internalization (**C**) was however significantly reduced with these macropinocytotic inhibitors. NS P>0.1, hA vs. hA/inhibitors, ^##^P<0.01, dextran vs. dextran/inhibitors, n = 9. Significance established by ANOVA followed by Dunnett-Square test. Bar 10µm.

**Figure 11:**
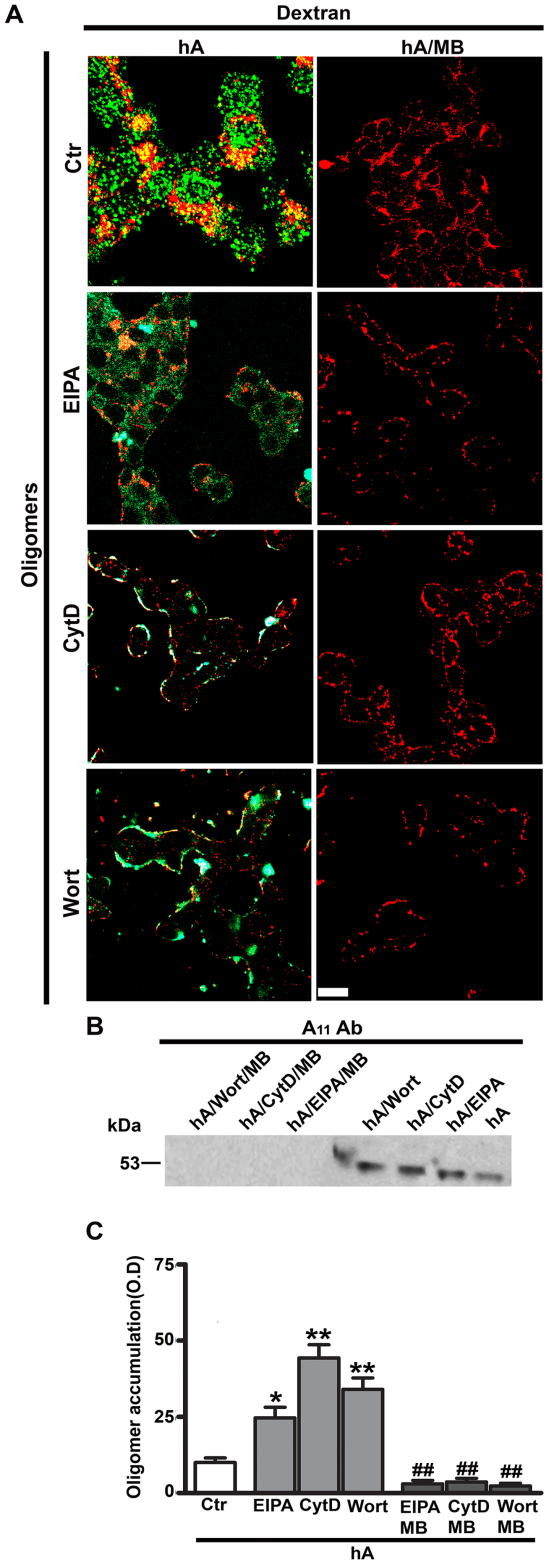
Inhibition of macropinocytosis increases amylin oligomerization in human islets. Cells were first treated with macropinocytotic inhibitors (EIPA, CytD or Wort) for 1 hour followed by addition of human amylin (10 µM) either in the absence or presence of MB for 24 hours at 37°C. Dextran was finally added for an additional 30 minutes. (**A**) Immunoconfocal microscopy revealed a significant increase in the PM accumulation of both amylin oligomers (green), detected with A11 antibody and dextran (red) when treated with these inhibitors as compared to the controls (human amylin-treated cells) (left panel). However, in the presence of MB (100 µM), only dextran but not oligomer accumulation was detected on the PM (right panel). Bar 10µm. Extracellular content of amylin oligomers was analyzed by (**B**) western blot and (**C**) densitometry. Inhibition of macropinocytosis stimulated oligomer accumulation in the culturing medium, which was reversed by addition of MB. **P<0.01, hA vs. hA/inhibitors, n = 3, ANOVA followed by Dunnett-Square test and ^##^P<0.01, hA/inhibitors vs. hA/inhibitors/MB, n = 3, ANOVA followed by Newman-Keul post hoc test.

Next, we sought to determine if macropinocytosis of human amylin is specific for RIN-m5F cells or it may also account for uptake of amylin monomers and oligomers in human pancreatic islet cells (Figure 10–11). Confocal microscopy revealed a significant accumulation (72±3%) of monomers on the PM of dissociated human islet cells following incubation with amylin (Figure 10A–B). Internalization of monomers in human islets remained unchanged in the presence of specific macropinocyotic inhibitors (Figure 10A–B). However, dextran internalization was significantly reduced to 9–12% by these inhibitors with a concomitant increase in its PM accumulation (Figure 10A, C). A11 positive-amylin oligomers entered islet cells by macropinocytosis as evident by a marked decrease in their internalization by EIPA, CytD or Wort (Figure 11A, left panel). The stimulatory effect of these macropinocytotic inhibitors on amylin oligomer accumulation on the PM was completely blocked by addition of MB (Figure 11A, right panel) while dextran turnover was unaffected by MB (Figure 11A, right panel). Western blot analyses followed by densitometry (Figure 11B–C) confirmed a 22–41% increase in accumulation of intermediate sized amylin oligomers in the culture medium of human islets treated with macropinocytotic inhibitors as compared to cells incubated with human amylin only. As for PM, MB completely abolished oligomer accumulation in the medium (Figure 11B–C).

### Inhibition of Macropinocytosis Stimulates Human Amylin Toxicity in Pancreatic Cells

**Figure 12:**
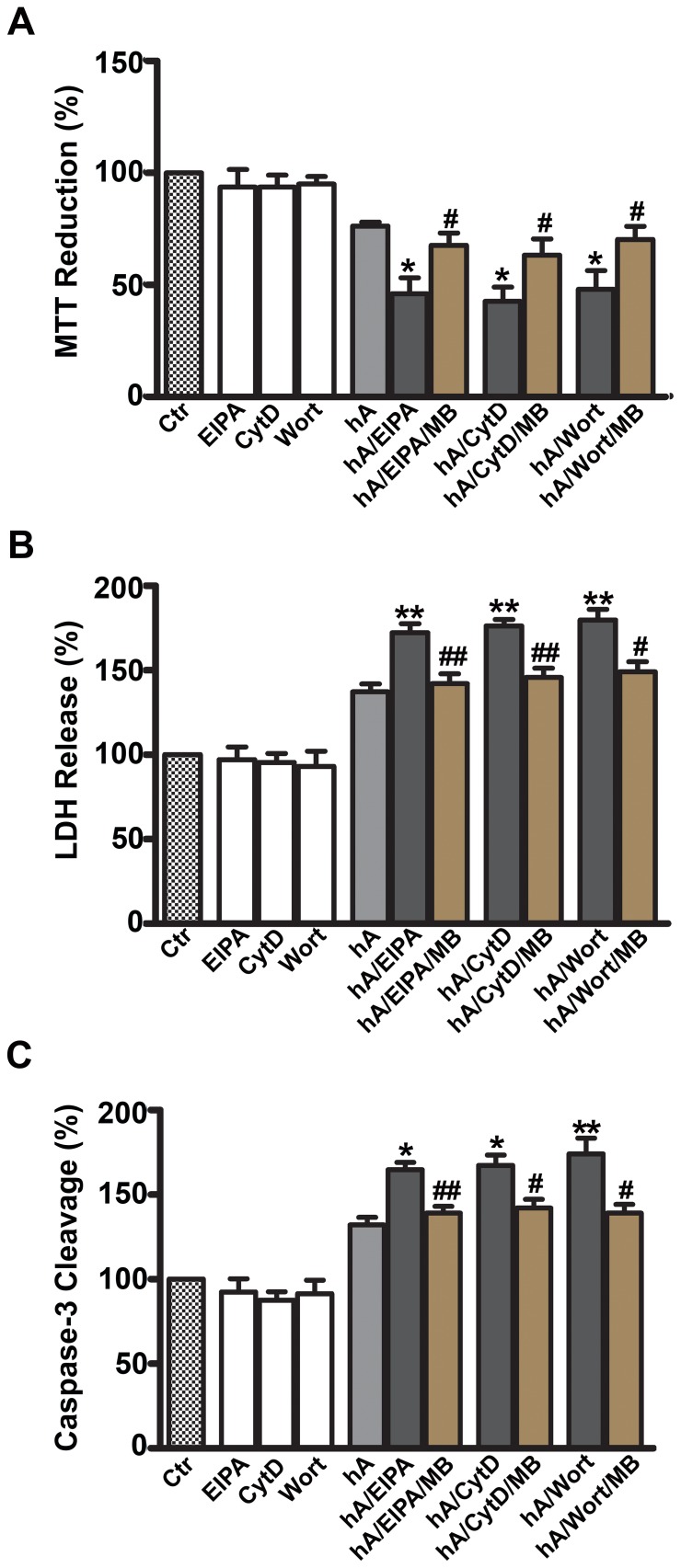
Inhibition of macropinocytosis augments human amylin toxicity in RIN-m5F cells. The effects on cell viability were assessed with MTT reduction (**A**), LDH release (**B**) and Caspase-3/7 cleavage (**C**). Inhibition of macropinocytosis by EIPA, CytD or Wort enhanced human amylin toxicity relative to the control cells (human amylin alone), which in turn was significantly reversed by adding MB. *P<0.05, **P<0.01, hA vs. hA/inhibitors, n = 9 ANOVA followed by Dunnett-Square test and ^#^P<0.05, ^##^P<0.01, hA/inhibitors vs. hA/inhibitors/MB, n = 9, ANOVA followed by Newman-Keul post hoc test.

**Figure 13:**
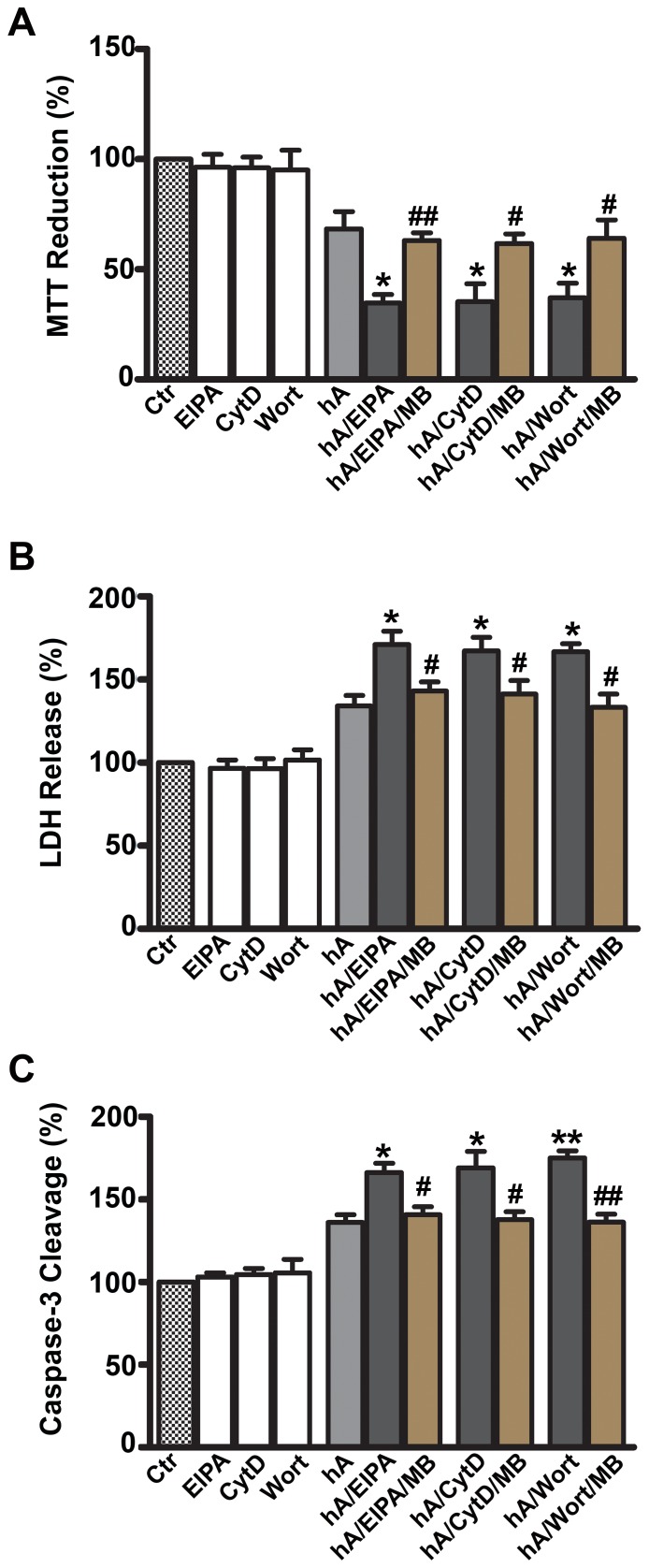
Inhibition of macropinocytosis potentiates amylin toxicity in human islets. The effects on cell viability were assessed with MTT reduction (**A**), LDH release (**B**) and Caspase-3/7 cleavage (**C**) cytotoxic assays. Inhibition of macropinocytosis by its inhibitors (EIPA, CytD or Wort) enhanced amylin toxicity relative to the control cells (human amylin only), which in turn was reversed by adding MB to these cells. *P<0.05, **P<0.01, hA vs. hA/inhibitors, n = 9 ANOVA followed by Dunnett-Square test and ^#^P<0.05, ^##^P<0.01, hA/inhibitors vs. hA/inhibitors/MB, n = 9, ANOVA followed by Newman-Keul post hoc test.

Since macropinocytosis is implicated in the clearance of cytotoxic amyloid oligomers in the pancreatic cells and other cell types [47], we investigated the inhibition of macropinocytosis with respect to amylin toxicity in RIN-m5F cells (Figure 12) and human islets (Figure 13). Amylin alone induced a significant 30% decrease in mitochondrial activity (Figure 12A), increased LDH release (leakage) by 33% from cells (Figure 12B) and stimulated apoptotic caspase-3 activity by 34% (Figure 12C) relative to the controls (set at 100%). A similar detrimental effect of human amylin on viability was demonstrated in human islets as evident by a 32±8% decrease in mitochondrial activity (Figure 13A), a 34±6% increase in LDH release (Figure 13B) and a 36±5% increase in apoptotic caspase-3 activity respective of the controls (Figure 13C). We next determined the modulatory effects of the macropinocytotic inhibitors (EIPA, CytD or Wort) in RIN-m5F cells (Figure 12A) and human islets (Figure 13A). Although, these inhibitors were not toxic to the cells on their own, they augmented the inhibitory effect of human amylin on cell’s metabolic activity (MTT reduction) by 30–34% in both cell types (Figure 12A, 13A). This increase in amylin toxicity was reversed by MB that also blocked oligomer formation and hence, their extracellular accumulation. Because the MTT assay reflects both reversible and irreversible patho-physiological events that may or may not lead to cell death [4], we verified the effect of the same inhibitors on amylin toxicity using LDH release assay (Figure 12B, 13B). EIPA, CytD or Wort stimulated 32–37% increase in human amylin-evoked LDH release in RIN-m5F cells (Figure 12B) and human islet cells (Figure 13B). Cell viability was restored by the addition of anti-oligomer inhibitor, MB [4], [74]. Macropinocytotic inhibitors increased human amylin evoked apoptosis in both cell types by 30–40% revealed by the caspase3/7 apoptotic assay, which again was reversed by MB (Figure 12C, 13C). This suggests that an increase in extracellular oligomer content is toxic to cells, which is augmented by impaired macropinocytosis.

## Discussion

Although previous studies have demonstrated a causal connection between amylin oligomerization/aggregation and toxicity in pancreatic cells [4], [11], [26]–[33], little is known about the cellular factors and mechanisms that may protect these cells from amylin insult. In this current study, we investigated the role of AM-R and endocytosis in amylin monomer and oligomer internalization and how they impact amylin toxicity in pancreatic cells.

Western blot and immunoconfocal microscopy revealed that RIN-m5F β-cells and human islets predominantly express type-1 and type-2 AM-R respectively. Three different AM-R isoforms are found to be expressed in many cells and tissues [13], [14], [19], [20]; however their roles in amylin functions remain obscure. Notably, human amylin at concentrations found in the serum (10^−10^–10^−8^ M), inhibits insulin release from pancreatic cells, thereby maintaining glucose homeostasis [6], [8]–[10], [75]. However, the mechanism and the receptor mediating this inhibitory effect of human amylin still remain elusive. Our results indicate that human amylin when added at low (1–100 nM) concentrations stimulated AM-R re-cycling to the PM, and inhibited insulin secretion from pancreatic cells in a dose dependent manner, the effect of which was reversed by selective AM-R antagonist, AC-187, indicating involvement of AM-R in this process. Consistent with the formation of a functional amylin-AM-R complex on the pancreatic cell PM, AC-187, specifically blocked the uptake of human amylin only at low (100 nM) but not high (10 µM) concentrations. When used within this concentration range, AC-187 is several fold more potent an antagonist of type-1 and type-3 AM-R over CT-R [15].

In addition to its physiological effect, the possible role of AM-R in amylin toxicity is also unclear, and hence was investigated in the current study. In contrast to the physiological effect of human amylin achieved at low nM range, viability studies indicate that amylin’s toxicity is not mediated by AM-R in either of the pancreatic cells as high (>2 µM) concentrations were required to induce apoptosis in these cells. Furthermore, addition of AC-187 failed to alter amylin toxicity in pancreatic cells, although the same concentration (100 nM) of this antagonist effectively blocked amylin uptake and completely reversed the inhibitory effect of human amylin on insulin release. Previous studies indicated that expression of type-3 but not type-1 and type-2 AM-R in HEK293 cells and human fetal neurons is required for the toxic actions of human amylin and β-amyloid [62], [65]. AM-R antagonists, AC-187 and AC-253, when used at high (µM) concentrations and for extensive pre-incubation times, effectively reversed human amylin and β-amyloid toxicity in these neuronal cells [62], [63], [65]. Interestingly, our western blot analysis revealed that type-1 and type-2 but not type-3 are major AM-R isoforms expressed in pancreatic cells. Thus, different cellular backgrounds and/or expression of distinct AM-R subtypes could account for the different toxic mechanisms of human amylin in pancreatic and neuronal cells.

Besides AM-R, we also investigated other cellular factors implicated in internalization of human amylin in pancreatic cells. Our results indicate that during an initial phase (within the first hour), the majority of human amylin monomers and oligomers entered pancreatic cells via macropinocytosis. This conclusion is supported by the finding, that under control conditions, both human amylin monomers and oligomers co-localized with dextran, a marker of macropinosomes. Section analysis of confocal micrographs revealed that average size of these compartments was between 1–2 µm, within the size range of macropinososmes [46], [47], [67], [76]. In addition, macropinocytotic inhibitors, EIPA, CytD or Wort, significantly reduced amylin monomer and oligomer uptakes, further implicating macropinocytosis. Interestingly, macropinocytosis of human amylin and dextran was found to be dynamin-independent. The above findings are in agreement with the previous study showing that the macropinocytotic pathway is involved in the uptake of the brain β-amyloid peptide by microglia cells [47]. Immunoconfocal microscopy analysis also revealed that a small fraction (12–13%) of monomers and oligomers was internalized in pancreatic cells at 4°C, indicating a non-endocytotic (translocation) mechanism. Our results are in partial agreement with a previous study showing translocation of human amylin in rat insulinoma, INS1 cells [50]. However, under our experimental conditions, endocytosis but not translocation was the predominant uptake mechanism for human amylin in RIN-m5F cells and human islets. The propensity of human amylin to insert into synthetic and native membranes [23], [36], [50], [77], along with its amphiphilic and strong cationic character, may explain its ability to translocate across the PM by a non-endocytotic mechanism as observed in our study and originally reported by the Miranker group [50]. In this respect, human amylin mimics the membrane permeating actions of cell penetrating peptides, CPPs [50]. CPPs are diverse classes of cationic peptides that like amylin are rich in arginine and lysine residues [50], [58], [59], [78]. Two distinct internalization pathways, endocytotic and non-endocytotic have been reported for CPPs, the preponderance of which depends on peptide sequence, peptide concentration, peptide/cell ratio and membrane composition [50], [58], [59], [78]. Thus, the differences in internalization mechanisms for human amylin may be due to varying peptide/cell ratio and/or different cell types used in the two studies [50]. That peptide/cell ratio may determine the mode of amylin internalization is further suggested by our confocal microscopy studies showing that, during early phase when human amylin concentrations are high, AM-R independent macropinocytosis mediated amylin monomer and oligomer uptake, which did not require clathrin or dynamin.

In contrast to the early phase, the late phase of human amylin monomer internalization in pancreatic cells (measured at 24 hours) required clathrin. This is in agreement with our previous study showing that amylin monomer internalization was blocked by the clathrin pharmacological inhibitor, chlorpromazine [4]. We found amylin in the culture medium of pancreatic cells throughout the duration of studies (0–24 hours) and observed its gradual disappearance from the medium. The marked drop in amylin concentration in the culture medium at the late stage (12–24 hours), could explain a shift from non-saturable, receptor independent macropinocytosis to a saturable, CME. Interestingly, macropinocytosis also mediated the uptake of amylin oligomers. Consistent with our findings, fluid phase endocytosis was also implicated in the uptake of β-amyloid derived-oligomers in neural cells [47]. The endocytotic hypothesis of amylin transport into pancreatic cells is further supported by EM studies revealing PM invaginations at the site of amylin accumulation and the association of internalized amylin with clathrin-derived endocytotic vesicles [49]. However, like human amylin, β-amyloid could also translocate by a non-endocytotic mechanism [52], [57], demonstrating plasticity in internalization mechanisms for amyloid peptides.

Thus, our results suggest that endocytosis, in particular macropinocytosis, plays an important role in the uptake of human amylin monomers and oligomers in pancreatic cells. Our results show that pharmacological inhibition of macropinocytosis prevented internalization of A11 positive amylin oligomers, in turn stimulating their accumulation on the PM correlated with a rise in amylin toxicity. This increase in amylin oligomer formation and accumulation was completely blocked with methylene blue, a specific amylin oligomer inhibitor [4], [74], [79], which in turn reversed human amylin toxicity. These results implicate macropinocytosis as a major pathway for clearance of human amylin monomers and cytotoxic oligomers.

In summary, our results indicate that AM-R and endocytosis play pivotal roles in the uptake of human amylin monomers and oligomers in pancreatic cells. The internalization was both time and concentration dependent, both factors dictating the mechanism of amylin entry into these cells. Although, some amylin was able to translocate by a non-endocytotic mechanism, the majority of the monomers and oligomers followed macropinocytosis. The significant increase in amylin toxicity in macropinocytosis-impaired cells suggests a cyto-protective mechanism operating in pancreatic cells.

## Supporting Information

Figure S1 **Amylin receptor-dependent and -independent mechanisms of human amylin internalization in human islets.** Cells were incubated with 100 nM or 10 µM human amylin either in the presence or absence of the AM-R antagonist, AC-187 (1–100 nM) for 24 hours. **(A)** Confocal microscopy and whole cell analysis revealed that when low concentration (100 nM) was used, amylin monomer internalization was significantly inhibited with increasing concentrations of AC-187 (top panel, graph). Monomer uptake at high (10 µM) was unchanged with increasing concentrations of AC-187 (bottom panel, graph). **(B)** Similarly, no change in cellular distributions of amylin oligomers, formed at high (10 µM) was observed in the presence of AC-187. **P<0.01, hA 100 nM vs. hA 100 nM/10 nM AC187, **^##^**P<0.01, hA 100 nM vs. hA 100 nM/100 nM AC187, NS P>0.1, hA 100 nM vs. hA 100 nM/1 nM AC187 and NS P>0.1, hA 10 µM vs. hA 10 µM/treatments, n = 9. Significance established by ANOVA followed by Dunnett-Square test. Bar 5µm.
(TIF)

Figure S2 **Two types of amylin receptor are expressed in RIN-m5F cells and human islets. (A)** Western blot shows expression of CT-R and two RAMPs isoforms RAMP_1_ in human islets (H) and RAMP_2_ in RIN-m5F cells (R). **(B)** Immunoconfocal microscopy analysis revealed expression and location of RAMP_2_ (green)/CT-R (red) in RIN-m5F cells (top panel) and RAMP_1_ (green)/CT-R (red) in human islet cells (bottom panel). Bar 10µm. **(C)** The inhibitory effect of human amylin on glucose-evoked insulin release from human islets was reversed by addition of AM-R antagonist, AC-187, indicating an AM-R mediated process. Intact human islets were exposed to glucose (glc), human amylin (hA) and/or AC-187 for 30 minutes and insulin content in the samples was analyzed by ELISA. Data was normalized to total protein content in samples. ^#^P<0.05, 5 mM Glc vs. 16 mM Glc, n = 6, unpaired student’s t-test; *P<0.05, **P<0.01, control vs. hA 0.2–100 nM; and ^&^P<0.05, hA 100 nM vs. hA 100 nM +AC-187 100 nM, n = 6.Significance established ANOVA followed by Dunnett-Square test.
(TIF)

Figure S3 **Amylin toxicity is amylin receptor independent in human islets.** MTT reduction **(A)**, LDH release **(B)** and Caspase-3/7 cleavage **(C)** studies demonstrated that toxicity of 10 µM human amylin is independent of its receptor as the toxicity remained unchanged in the presence of increasing concentrations of the AM-R antagonist, AC-187. NS P>0.1, hA vs. hA/treatments, n = 9. Significance established by ANOVA followed by Dunnett-Square test.
(TIF)

Figure S4 **Initial entry of amylin monomers and oligomers is through dynamin-independent macropinocytosis in RIN-m5F cells.** Cells were treated with EIPA, CytD, Wort or Dyn for 1 hour followed by human amylin (green) (10 µM) for an additional hour at 37°C. Dextran (red) was finally added for 30 minutes. **(A)** Confocal microscopy (top panel) revealed a significant reduction in internalization and increase in PM accumulation of amylin monomers (green) and dextran (red) in the presence EIPA, CytD or Wort but not Dyn when compared to controls. Macropinocytotic inhibitors also prevented internalization of amylin oligomers within the first hour **(A, bottom panel)**. Bar 10µm. Amylin monomers **(B)** and oligomers **(C)** partially co-localized with dextran under control conditions. Following treatments with macropinocytotic inhibitors but not with Dyn, there was a significant decrease in their respective co-localization with dextran. **P<0.01, hA vs. hA/inhibitors, NS P>0.1, hA vs. hA/Dyn, n = 9. Significance established by ANOVA followed by Dunnett-Square test.
(TIF)

Figure S5 **Amylin monomer internalization is independent of clathrin and dynamin at 1 hour in RIN-m5F cells.** Cells were treated with Dyn or Chl for 1 hour followed by human amylin (green) (10 µM) for an additional 1 hour at 37°C. In parallel, cells were incubated with human amylin (10 µM) for 1 hour at 4°C. CTX (red) and Trf (blue) were finally added for 30 minutes at 37°C or 4°C. Immunoconfocal microscopy **(A)** and whole cell analysis **(B–D)** demonstrated no noticeable difference in cellular distributions of monomers **(B)** when treated with Dyn or Chl. However, lowering temperature to 4°C blocked monomer internalization as well as CTX and Trf **(B–D)**. Arrowheads and arrows denote β-cells with internalized and PM associated amylin monomers, respectively. NS P>0.1, hA, vs. hA/inhibitors and **P<0.01, hA vs. hA/4°C, n = 9. CTX uptake **(C)** was unchanged by Chl but was significantly reduced in the presence of Dyn or 4°C, in turn causing an accumulation of CTX on cell PM. ^##^P<0.01, CTX vs. CTX/dyn, **P<0.01, CTX vs. CTX/4°C and NS P>0.1, CTX vs. CTX/Chl, n = 9. Internalization of Trf **(D)** was however significantly decreased by Chl or Dyn along with a marked inhibition observed at 4°C.^ ##^P<0.01, Trf vs. Trf/dyn, **P<0.01, Trf vs. Trf/4°C and @@ P<0.01, Trf vs. Trf/Chl, n = 9. Significance established by ANOVA followed by Dunnett-Square test. Bar 10µm.
(TIF)

Figure S6 **Initial entry of amylin oligomers is independent of clathrin and dynamin in RIN-m5F cells.** Cells were treated with Dyn or Chl for 1 hour followed by human amylin (green) (10 µM) for an additional 1 hour at 37°C. Additionally, cells were also incubated with human amylin (10 µM) for 1 hour at 4°C. CTX (red) and Trf (blue) were finally added for 30 minutes at 37°C or 4°C. Confocal microscopy **(A)** and whole cell analysis **(B–D)** show no significant change in the cellular distributions of amylin oligomers **(B)** when treated with Dyn or Chl. However, there was a significant decrease in internalization and an increase in PM accumulation of these molecular forms at 4°C. Arrowheads and arrows represent β-cells with internalized and PM associated amylin monomers, respectively. NS P>0.1, hA vs. hA/inhibitors and **P<0.01, hA vs. hA/4°C, n = 9. CTX internalization **(C)** was unaffected by Chl but was significantly reduced in the presence of Dyn or 4°C, in turn causing an increase in PM CTX accumulation. ^##^P<0.01, CTX vs. CTX/dyn, **P<0.01, CTX vs. CTX/4°C and NS P>0.1, CTX vs. CTX/Chl, n = 9. Internalization of Trf **(D)** was however significantly decreased by either Chl or Dyn along with a marked inhibition observed at 4°C.^ ##^P<0.01, Trf vs. Trf/dyn, **P<0.01, Trf vs. Trf/4°C and @@ P<0.01, Trf vs. Trf/Chl, n = 9. Significance established by ANOVA followed by Dunnett-Square test. Bar 10µm.
(TIF)

Figure S7 **Late entry of amylin oligomers but not monomers is through dynamin-independent macropinocytosis in RIN-m5F cells.** Cells were either treated with EIPA, CytD, and Wort for 1 hour or transfected with dynamin mutant form, DN dyn1K44A for 16–18 hours followed by human amylin (green) (10 µM) incubation for an additional 24 hours at 37°C. Dextran (red) was finally added for 30 minutes. **(A)** Immunoconfocal microscopy revealed no significant change in the cellular distributions of amylin monomers (top panel) in the presence of EIPA, CytD, Wort or DN dyn1K44A when compared to controls. On the contrary, dextran internalization was completely blocked with these macropinocytotic inhibitors but not with DN dyn1K44A **(A, top panel)**. Marked inhibition in internalization of amylin oligomers and dextran was observed following treatments with EIPA, CytD or Wort but not with DN dyn1K44A **(A, bottom panel)**. Bar 10µm. **(B)** Amylin monomers at 24 hours did not co-localize with dextran either in the absence or presence of macropinocytotic inhibitors and dynamin mutant construct. NS, P>0.1, hA vs. hA/treatments, n = 9. **(C)** Oligomers partially co-localized with dextran under control conditions. Following treatments with EIPA, CytD or Wort but not with DN dyn1K44A, there was a significant decrease in their respective co-localization. **P<0.01, hA vs. hA/inhibitors and NS P>0.1, hA vs. hA/dyn1K44A, n = 9. Significance established by ANOVA followed by Dunnett-Square test.
(TIF)

Figure S8 **Dynamics of amylin monomer turnover in pancreatic RIN-m5F cells.** Cells were incubated with 10 µM human amylin and amylin content in the extracellular medium analyzed over 24 hours. 50 µl of cell culturing medium was periodically collected and analyzed by western blot using human amylin specific antibody (hA Ab) that detected only monomers but not higher MW amylin-derived oligomers.
(TIF)

Figure S9 **Late phase of amylin monomer internalization requires clathrin in RIN-m5F cells.** Cells were transfected with 1µg of wild type (wt-AP180) or DN clathrin adaptor AP180 protein for 16–18 hours. Following transfections, cells were incubated with either 100 nM or 10 µM human amylin (green) for an additional 24 hours at 37°C. Cells were also treated with human amylin at the indicated concentrations for 24 hours at 4°C. CTX (red) and Trf (blue) were finally added for 30 minutes at 37°C or 4°C after incubating the cells with 10 µM human amylin. Confocal microcopy and whole cell analysis revealed a significant reduction in internalization and an increase in PM accumulation of amylin monomers at 100 nM **(A, B)** or 10 µM **(C, D)** when transfected with DN AP180CFLAG or when incubated at 4°C. In contrast, there was no change in their cellular distributions in wt-AP180 expressed cells and controls. NS P>0.1, hA vs. hA/wt-AP180, **P<0.01, hA vs. hA/AP180CFLAG and ^##^P<0.01, hA vs. hA/4°C, n = 9. CTX internalization **(C, E)** was however unchanged with DN AP180CFLAG expression as compared to a marked inhibition in internalization of Trf **(C, F)**. CTX and Trf internalization were blocked at 4°C in turn causing significant increases in their PM accumulations. NS P>0.1, CTX vs. CTX/wt-AP180, NS P>0.1, CTX vs. CTX/AP180CFLAG, ^##^P<0.01, CTX vs. CTX/4°C, NS P>0.1, Trf vs. Trf/wt-AP180, **P<0.01, Trf vs. Trf/AP180CFLAG and ^##^P<0.01 Trf vs. Trf/4°C, n = 9. Significance established by ANOVA followed by Dunnett-Square test. Bar 10µm.
(TIF)

Figure S10 **Late entry of amylin oligomers is independent of clathrin in RIN-m5F cells.** Cells were transfected with 1µg of wild type (wt-AP180) or DN clathrin adaptor AP180 protein, for 16–18 hours. Following this, cells were incubated with 10 µM human amylin (green) for an additional 24 hours at 37°C. In parallel, human amylin was incubated with cells for 24 hours at 4°C. CTX (red) and Trf (blue) were finally added for additional 30 minutes at 37°C or 4°C. Confocal microscopy **(A)** and whole cell analysis **(B–D)** revealed no significant change in cellular distributions of amylin oligomers **(B)** when transfected with wt-AP180 or DN AP180CFLAG respective to the controls (non-transfected cells). However, there was almost a complete block in internalization of these cytotoxic forms at 4°C. NS P>0.1, hA vs. hA/wt-AP180, NS P>0.1, hA vs. hA/AP180CFLAG and **P<0.01, hA vs. hA/4°C, n = 9. CTX internalization **(C)** was unaffected with DN AP180CFLAG expression as compared to a marked inhibition in internalization of Trf **(D)**. Both CTX **(C)** and Trf **(D)** internalization were further blocked at 4°C, causing an accumulation of these particles on the PM. NS P>0.1, CTX vs. CTX/wt-AP180, NS P>0.1, CTX vs. CTX/AP180CFLAG, **P<0.01, CTX vs. CTX/4°C, NS P>0.1, Trf vs. Trf/wt-AP180, ^##^P<0.01, Trf vs. Trf/AP180CFLAG and **P<0.01, Trf vs. Trf/4°C, n = 9. Significance established by ANOVA followed by Dunnett-Square test. Bar 10µm.
(TIF)

Figure S11 **Late phase of amylin oligomer internalization is independent of dynamin in RIN-m5F cells**. Cells were transfected with 1µg of the pcDNA3.1 empty vector construct or dynamin mutant, DN dyn1K44Afor 16–18 hours. Cells were then incubated with 10 µM human amylin (green) for additional 24 hours at 37°C. CTX (red) and Trf (blue) were finally added for additional 30 minutes. Confocal microscopy **(A)** and whole cell analyses **(B–D)** revealed no significant change in internalization and PM accumulation of amylin oligomers **(B)** when transfected with either pcDNA3.1 empty vector construct or DN dyn1K44A mutant construct. NS P>0.1, hA vs. hA/pcDNA3.1 and NS P>0.1, hA vs. hA/DN dyn1K44A, n = 9. CTX **(C)** and Trf **(D)** were prevented from internalizing these cells when incubated with DN dyn1K44A but not with the pcDNA3.1. NS P>0.1, CTX/Trf vs. CTX, Trf/pcDNA3.1 and **P<0.01, CTX/Trf vs. CTX, Trf/DN dyn1K44A, n = 9. Significance established by ANOVA followed by Dunnett-Square test. Bar 10µm.
(TIF)
