# Supplementary figures and images for: Distinct Internalization Pathways of Human Amylin Monomers and Its Cytotoxic Oligomers in Pancreatic Cells
Source: PLoS One. 2013 Sep 3;8(9):e73080. doi: 10.1371/journal.pone.0073080 (PMC3760900; doi:10.1371/journal.pone.0073080)

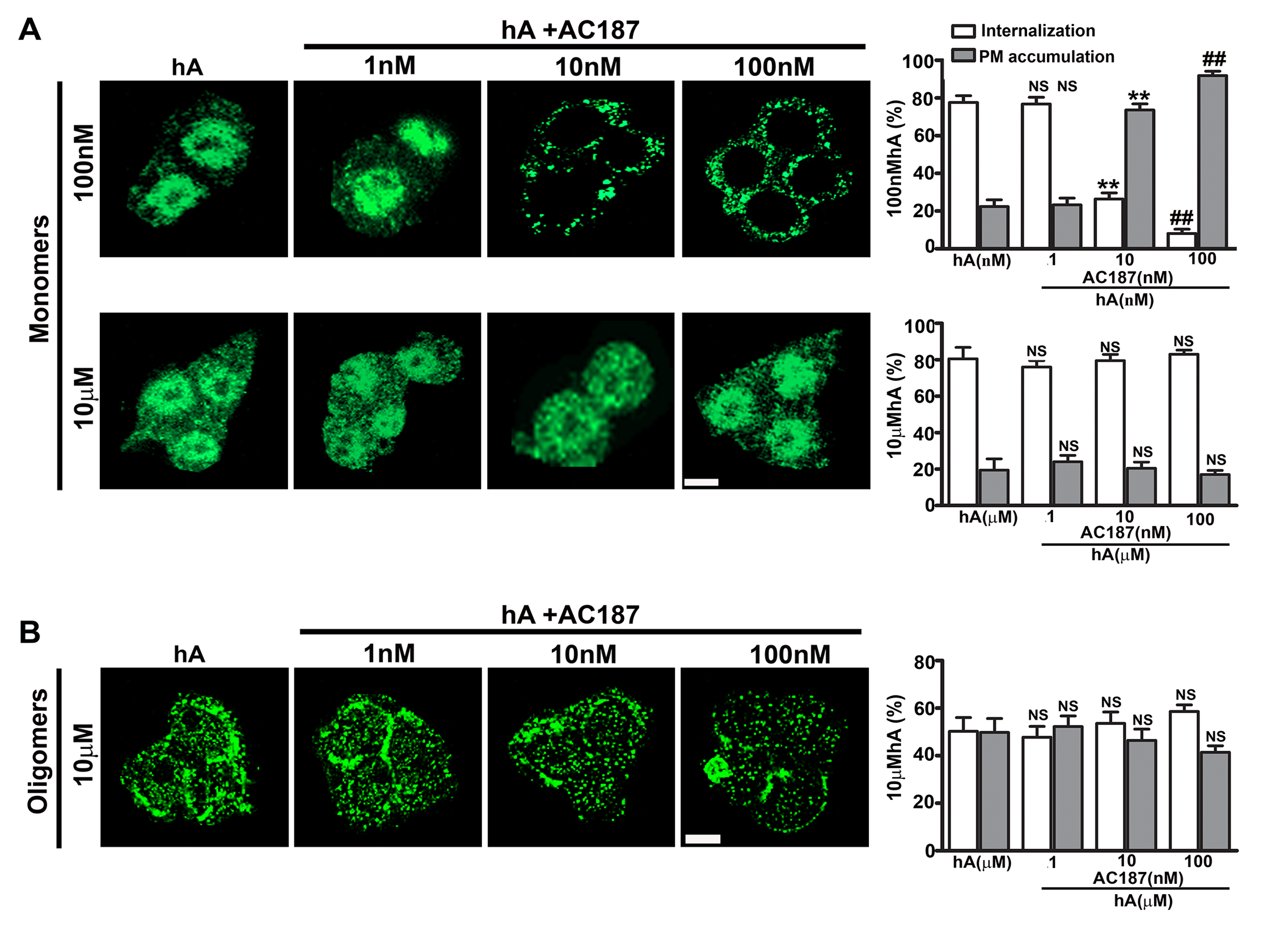

Supplement: Figure S1 — Amylin receptor-dependent and -independent mechanisms of human amylin internalization in human islets. Cells were incubated with 100 nM or 10 µM human amylin either in the presence or absence of the AM-R antagonist, AC-187 (1–100 nM) for 24 hours. (A) Confocal microscopy and whole cell analysis revealed that when low concentration (100 nM) was used, amylin monomer internalization was significantly inhibited with increasing concentrations of AC-187 (top panel, graph). Monomer uptake at high (10 µM) was unchanged with increasing concentrations of AC-187 (bottom panel, graph). (B) Similarly, no change in cellular distributions of amylin oligomers, formed at high (10 µM) was observed in the presence of AC-187. **P<0.01, hA 100 nM vs. hA 100 nM/10 nM AC187, ##P<0.01, hA 100 nM vs. hA 100 nM/100 nM AC187, NS P>0.1, hA 100 nM vs. hA 100 nM/1 nM AC187 and NS P>0.1, hA 10 µM vs. hA 10 µM/treatments, n = 9. Significance established by ANOVA followed by Dunnett-Square test. Bar 5µm. (TIF) [file pone.0073080.s001.tif]

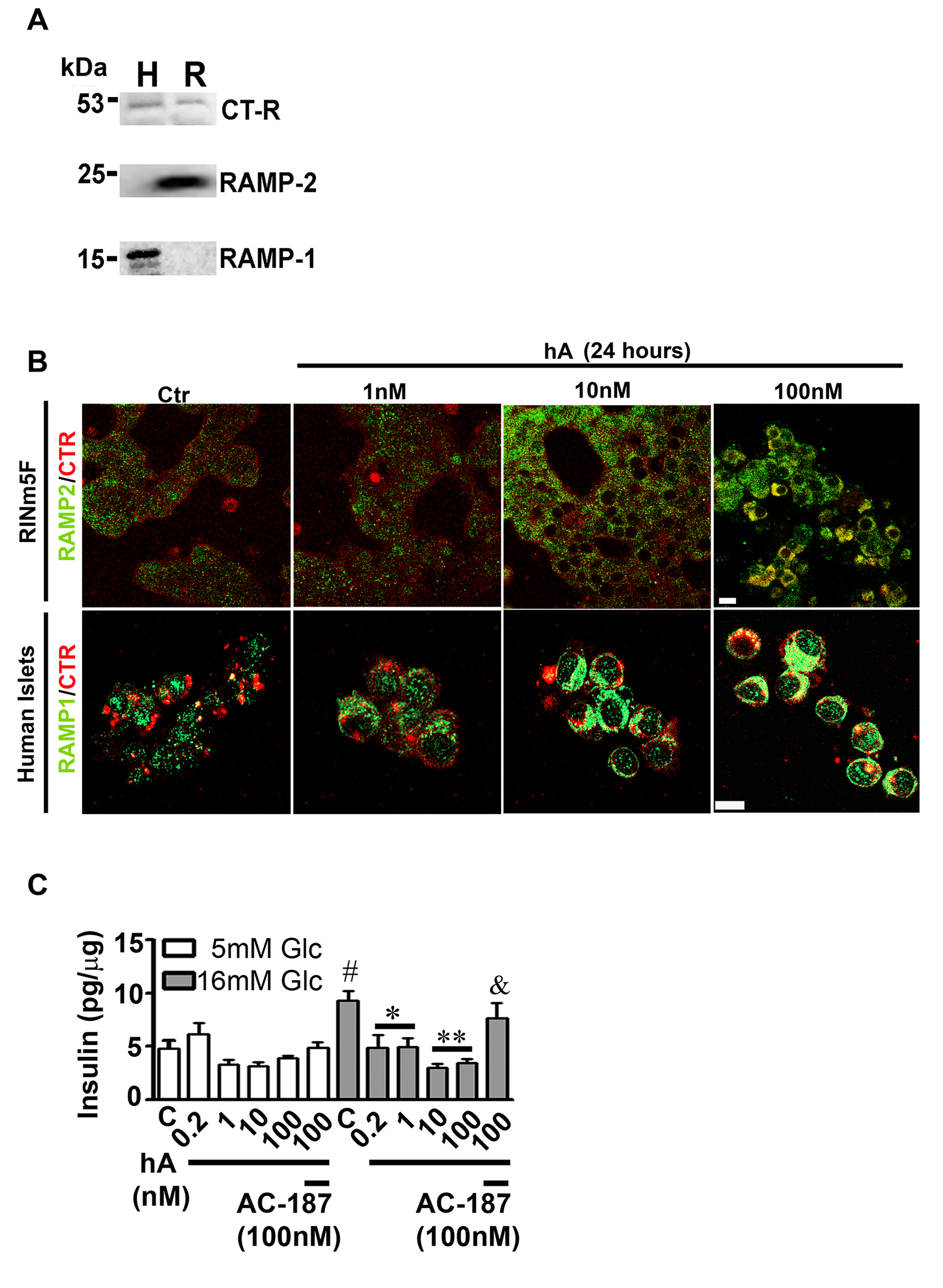

Supplement: Figure S2 — Two types of amylin receptor are expressed in RIN-m5F cells and human islets. (A) Western blot shows expression of CT-R and two RAMPs isoforms RAMP1 in human islets (H) and RAMP2 in RIN-m5F cells (R). (B) Immunoconfocal microscopy analysis revealed expression and location of RAMP2 (green)/CT-R (red) in RIN-m5F cells (top panel) and RAMP1 (green)/CT-R (red) in human islet cells (bottom panel). Bar 10µm. (C) The inhibitory effect of human amylin on glucose-evoked insulin release from human islets was reversed by addition of AM-R antagonist, AC-187, indicating an AM-R mediated process. Intact human islets were exposed to glucose (glc), human amylin (hA) and/or AC-187 for 30 minutes and insulin content in the samples was analyzed by ELISA. Data was normalized to total protein content in samples. #P<0.05, 5 mM Glc vs. 16 mM Glc, n = 6, unpaired student’s t-test; *P<0.05, **P<0.01, control vs. hA 0.2–100 nM; and &P<0.05, hA 100 nM vs. hA 100 nM +AC-187 100 nM, n = 6.Significance established ANOVA followed by Dunnett-Square test. (TIF) [file pone.0073080.s002.tif]

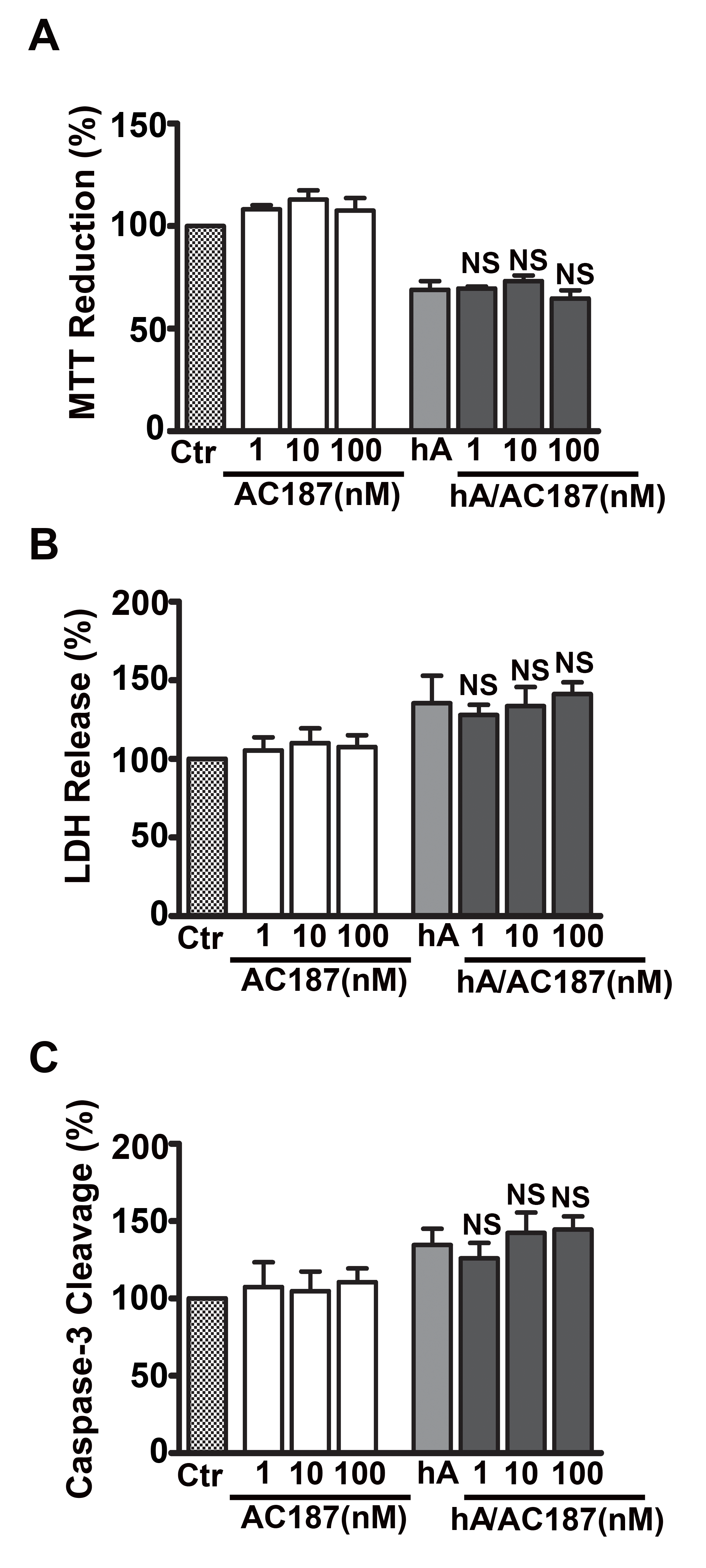

Supplement: Figure S3 — Amylin toxicity is amylin receptor independent in human islets. MTT reduction (A), LDH release (B) and Caspase-3/7 cleavage (C) studies demonstrated that toxicity of 10 µM human amylin is independent of its receptor as the toxicity remained unchanged in the presence of increasing concentrations of the AM-R antagonist, AC-187. NS P>0.1, hA vs. hA/treatments, n = 9. Significance established by ANOVA followed by Dunnett-Square test. (TIF) [file pone.0073080.s003.tif]

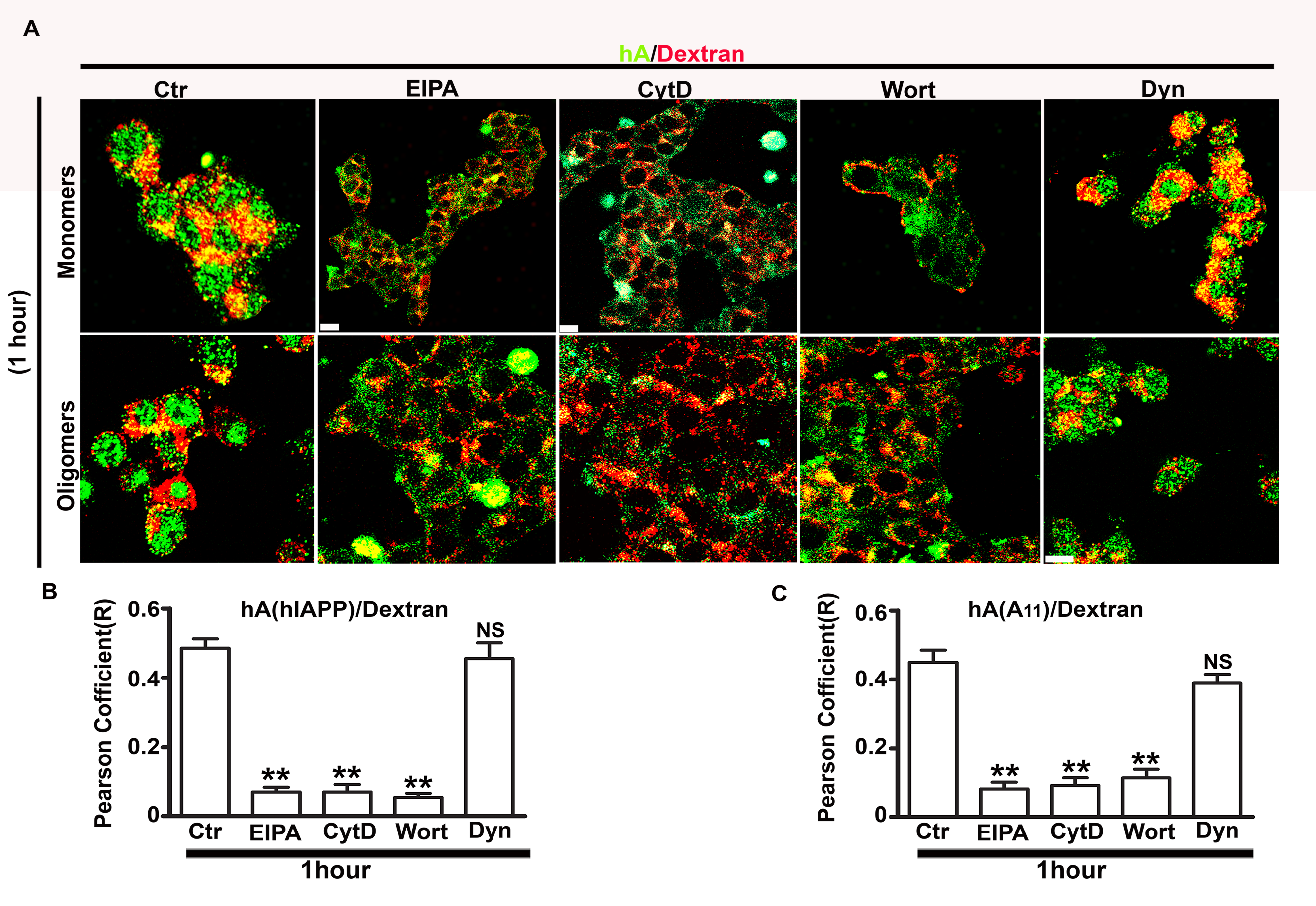

Supplement: Figure S4 — Initial entry of amylin monomers and oligomers is through dynamin-independent macropinocytosis in RIN-m5F cells. Cells were treated with EIPA, CytD, Wort or Dyn for 1 hour followed by human amylin (green) (10 µM) for an additional hour at 37°C. Dextran (red) was finally added for 30 minutes. (A) Confocal microscopy (top panel) revealed a significant reduction in internalization and increase in PM accumulation of amylin monomers (green) and dextran (red) in the presence EIPA, CytD or Wort but not Dyn when compared to controls. Macropinocytotic inhibitors also prevented internalization of amylin oligomers within the first hour (A, bottom panel). Bar 10µm. Amylin monomers (B) and oligomers (C) partially co-localized with dextran under control conditions. Following treatments with macropinocytotic inhibitors but not with Dyn, there was a significant decrease in their respective co-localization with dextran. **P<0.01, hA vs. hA/inhibitors, NS P>0.1, hA vs. hA/Dyn, n = 9. Significance established by ANOVA followed by Dunnett-Square test. (TIF) [file pone.0073080.s004.tif]

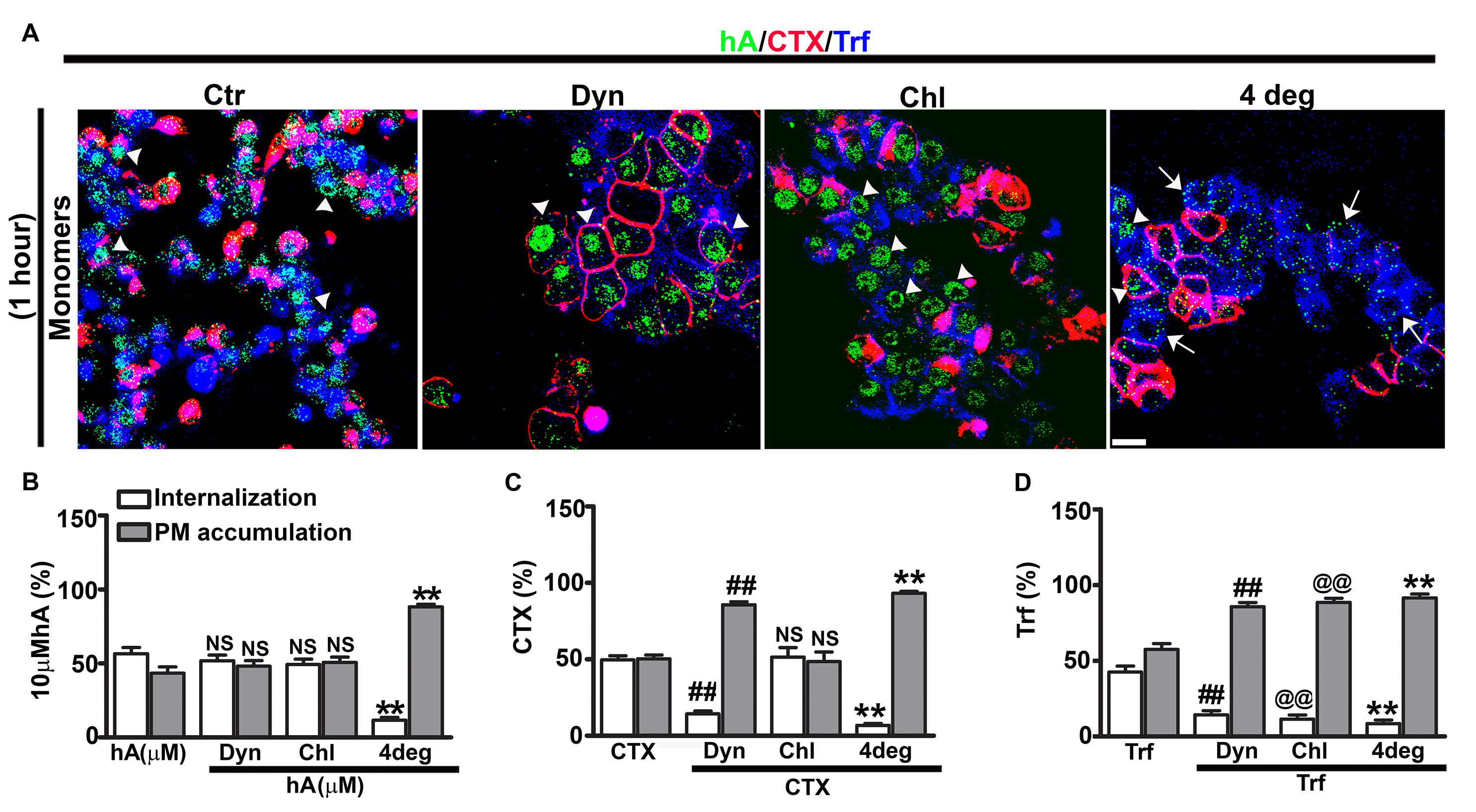

Supplement: Figure S5 — Amylin monomer internalization is independent of clathrin and dynamin at 1 hour in RIN-m5F cells. Cells were treated with Dyn or Chl for 1 hour followed by human amylin (green) (10 µM) for an additional 1 hour at 37°C. In parallel, cells were incubated with human amylin (10 µM) for 1 hour at 4°C. CTX (red) and Trf (blue) were finally added for 30 minutes at 37°C or 4°C. Immunoconfocal microscopy (A) and whole cell analysis (B–D) demonstrated no noticeable difference in cellular distributions of monomers (B) when treated with Dyn or Chl. However, lowering temperature to 4°C blocked monomer internalization as well as CTX and Trf (B–D). Arrowheads and arrows denote β-cells with internalized and PM associated amylin monomers, respectively. NS P>0.1, hA, vs. hA/inhibitors and **P<0.01, hA vs. hA/4°C, n = 9. CTX uptake (C) was unchanged by Chl but was significantly reduced in the presence of Dyn or 4°C, in turn causing an accumulation of CTX on cell PM. ##P<0.01, CTX vs. CTX/dyn, **P<0.01, CTX vs. CTX/4°C and NS P>0.1, CTX vs. CTX/Chl, n = 9. Internalization of Trf (D) was however significantly decreased by Chl or Dyn along with a marked inhibition observed at 4°C. ##P<0.01, Trf vs. Trf/dyn, **P<0.01, Trf vs. Trf/4°C and @@ P<0.01, Trf vs. Trf/Chl, n = 9. Significance established by ANOVA followed by Dunnett-Square test. Bar 10µm. (TIF) [file pone.0073080.s005.tif]

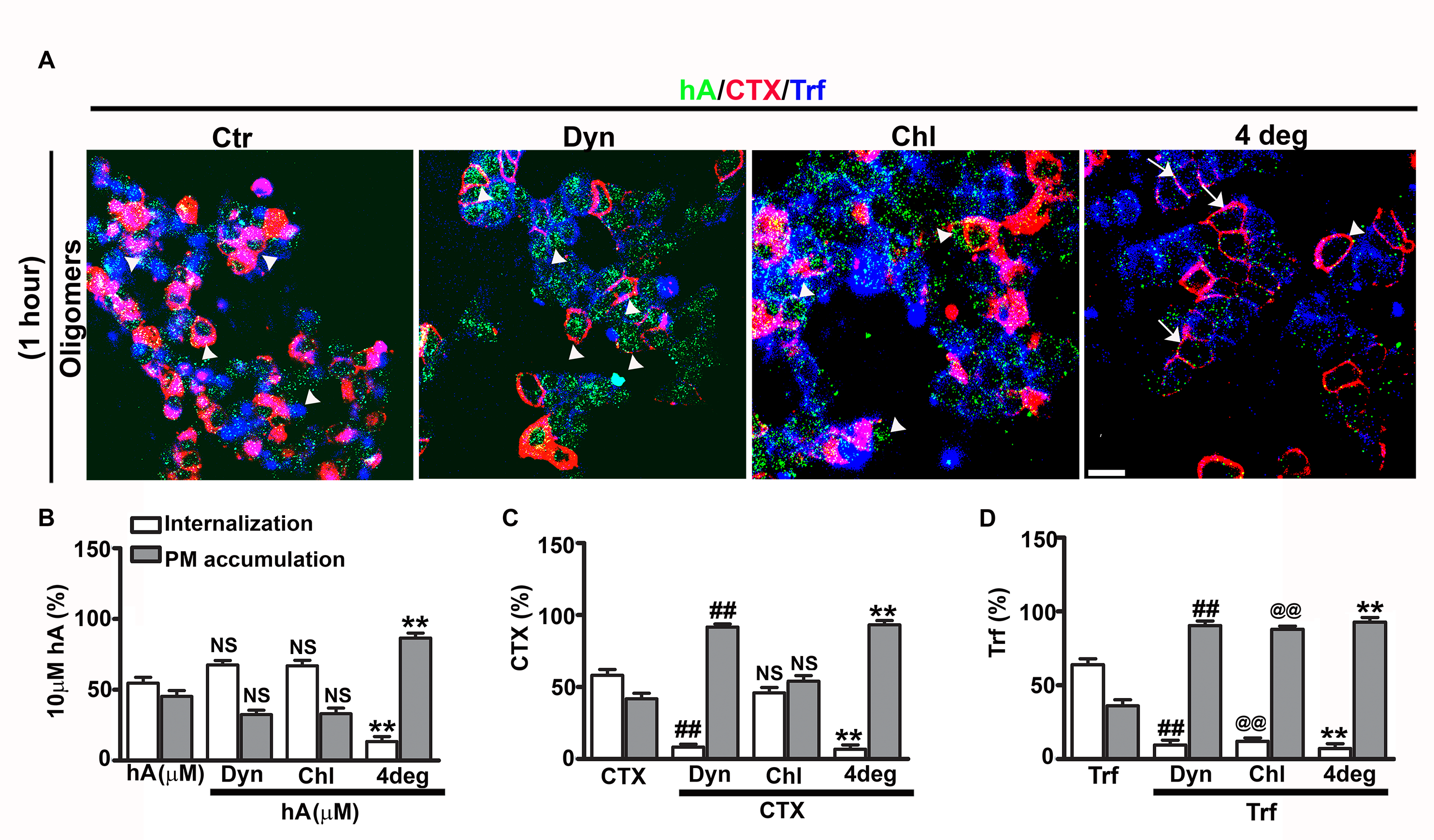

Supplement: Figure S6 — Initial entry of amylin oligomers is independent of clathrin and dynamin in RIN-m5F cells. Cells were treated with Dyn or Chl for 1 hour followed by human amylin (green) (10 µM) for an additional 1 hour at 37°C. Additionally, cells were also incubated with human amylin (10 µM) for 1 hour at 4°C. CTX (red) and Trf (blue) were finally added for 30 minutes at 37°C or 4°C. Confocal microscopy (A) and whole cell analysis (B–D) show no significant change in the cellular distributions of amylin oligomers (B) when treated with Dyn or Chl. However, there was a significant decrease in internalization and an increase in PM accumulation of these molecular forms at 4°C. Arrowheads and arrows represent β-cells with internalized and PM associated amylin monomers, respectively. NS P>0.1, hA vs. hA/inhibitors and **P<0.01, hA vs. hA/4°C, n = 9. CTX internalization (C) was unaffected by Chl but was significantly reduced in the presence of Dyn or 4°C, in turn causing an increase in PM CTX accumulation. ##P<0.01, CTX vs. CTX/dyn, **P<0.01, CTX vs. CTX/4°C and NS P>0.1, CTX vs. CTX/Chl, n = 9. Internalization of Trf (D) was however significantly decreased by either Chl or Dyn along with a marked inhibition observed at 4°C. ##P<0.01, Trf vs. Trf/dyn, **P<0.01, Trf vs. Trf/4°C and @@ P<0.01, Trf vs. Trf/Chl, n = 9. Significance established by ANOVA followed by Dunnett-Square test. Bar 10µm. (TIF) [file pone.0073080.s006.tif]

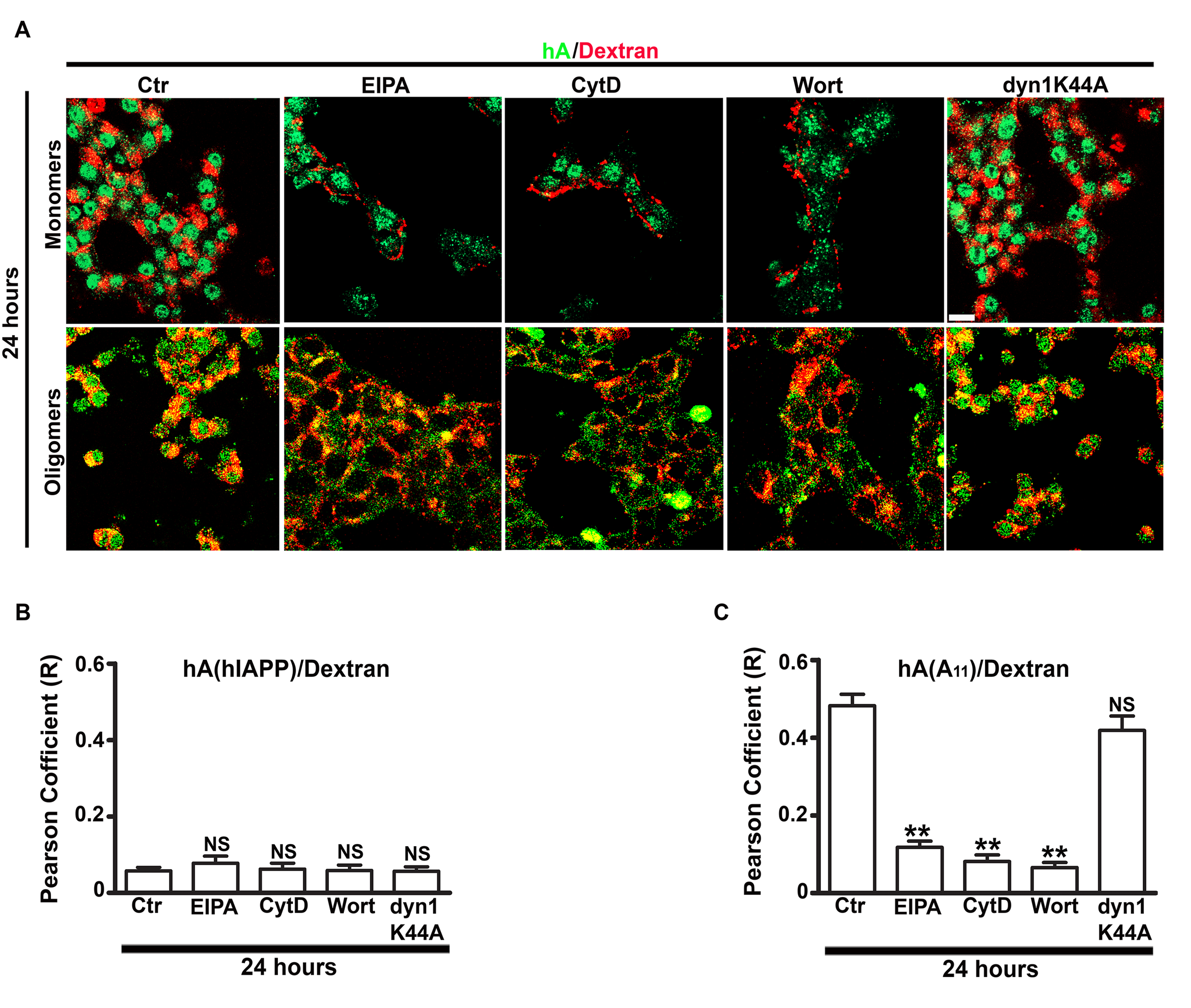

Supplement: Figure S7 — Late entry of amylin oligomers but not monomers is through dynamin-independent macropinocytosis in RIN-m5F cells. Cells were either treated with EIPA, CytD, and Wort for 1 hour or transfected with dynamin mutant form, DN dyn1K44A for 16–18 hours followed by human amylin (green) (10 µM) incubation for an additional 24 hours at 37°C. Dextran (red) was finally added for 30 minutes. (A) Immunoconfocal microscopy revealed no significant change in the cellular distributions of amylin monomers (top panel) in the presence of EIPA, CytD, Wort or DN dyn1K44A when compared to controls. On the contrary, dextran internalization was completely blocked with these macropinocytotic inhibitors but not with DN dyn1K44A (A, top panel). Marked inhibition in internalization of amylin oligomers and dextran was observed following treatments with EIPA, CytD or Wort but not with DN dyn1K44A (A, bottom panel). Bar 10µm. (B) Amylin monomers at 24 hours did not co-localize with dextran either in the absence or presence of macropinocytotic inhibitors and dynamin mutant construct. NS, P>0.1, hA vs. hA/treatments, n = 9. (C) Oligomers partially co-localized with dextran under control conditions. Following treatments with EIPA, CytD or Wort but not with DN dyn1K44A, there was a significant decrease in their respective co-localization. **P<0.01, hA vs. hA/inhibitors and NS P>0.1, hA vs. hA/dyn1K44A, n = 9. Significance established by ANOVA followed by Dunnett-Square test. (TIF) [file pone.0073080.s007.tif]

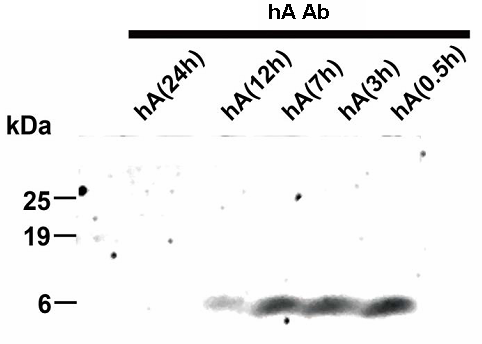

Supplement: Figure S8 — Dynamics of amylin monomer turnover in pancreatic RIN-m5F cells. Cells were incubated with 10 µM human amylin and amylin content in the extracellular medium analyzed over 24 hours. 50 µl of cell culturing medium was periodically collected and analyzed by western blot using human amylin specific antibody (hA Ab) that detected only monomers but not higher MW amylin-derived oligomers. (TIF) [file pone.0073080.s008.tif]

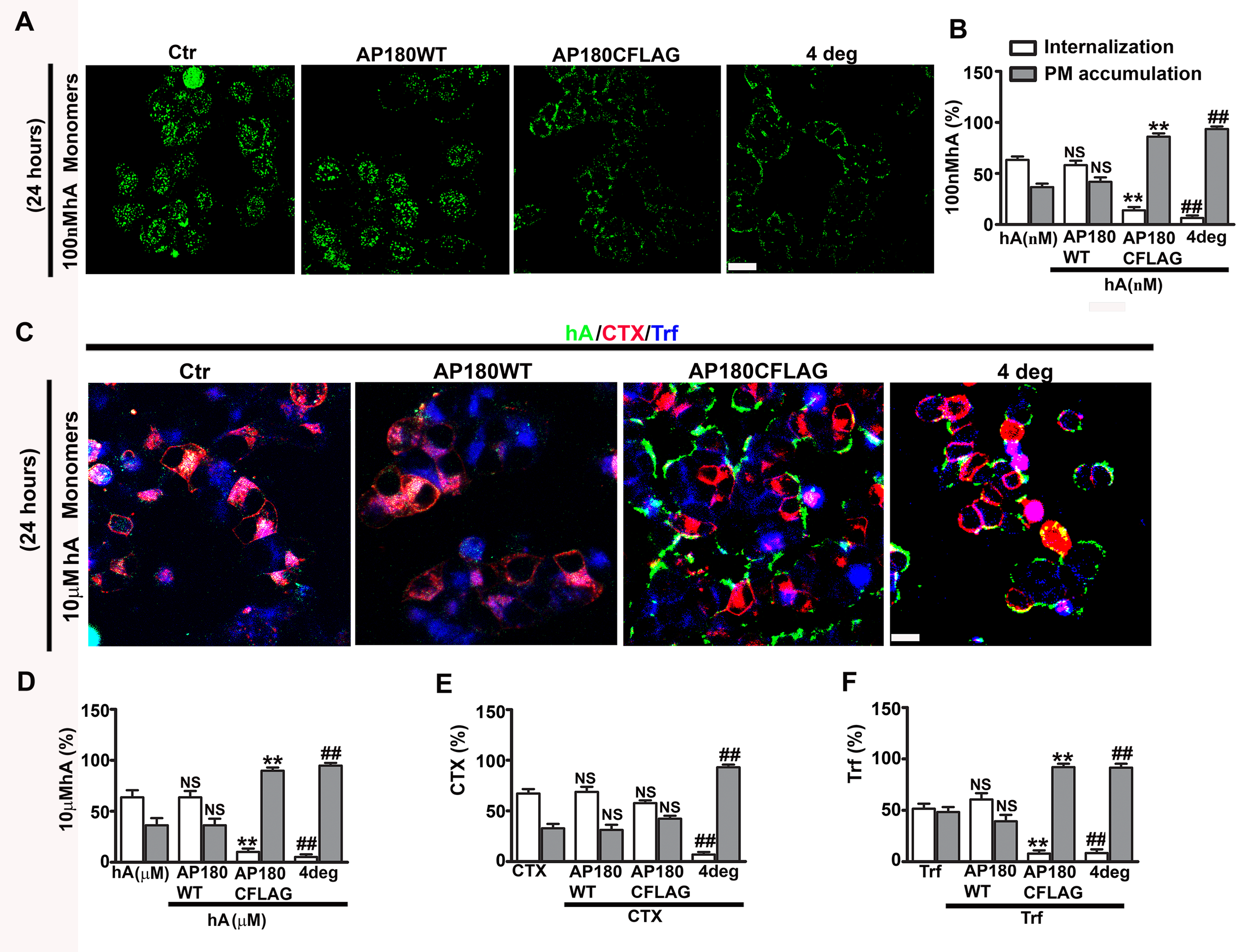

Supplement: Figure S9 — Late phase of amylin monomer internalization requires clathrin in RIN-m5F cells. Cells were transfected with 1µg of wild type (wt-AP180) or DN clathrin adaptor AP180 protein for 16–18 hours. Following transfections, cells were incubated with either 100 nM or 10 µM human amylin (green) for an additional 24 hours at 37°C. Cells were also treated with human amylin at the indicated concentrations for 24 hours at 4°C. CTX (red) and Trf (blue) were finally added for 30 minutes at 37°C or 4°C after incubating the cells with 10 µM human amylin. Confocal microcopy and whole cell analysis revealed a significant reduction in internalization and an increase in PM accumulation of amylin monomers at 100 nM (A, B) or 10 µM (C, D) when transfected with DN AP180CFLAG or when incubated at 4°C. In contrast, there was no change in their cellular distributions in wt-AP180 expressed cells and controls. NS P>0.1, hA vs. hA/wt-AP180, **P<0.01, hA vs. hA/AP180CFLAG and ##P<0.01, hA vs. hA/4°C, n = 9. CTX internalization (C, E) was however unchanged with DN AP180CFLAG expression as compared to a marked inhibition in internalization of Trf (C, F). CTX and Trf internalization were blocked at 4°C in turn causing significant increases in their PM accumulations. NS P>0.1, CTX vs. CTX/wt-AP180, NS P>0.1, CTX vs. CTX/AP180CFLAG, ##P<0.01, CTX vs. CTX/4°C, NS P>0.1, Trf vs. Trf/wt-AP180, **P<0.01, Trf vs. Trf/AP180CFLAG and ##P<0.01 Trf vs. Trf/4°C, n = 9. Significance established by ANOVA followed by Dunnett-Square test. Bar 10µm. (TIF) [file pone.0073080.s009.tif]

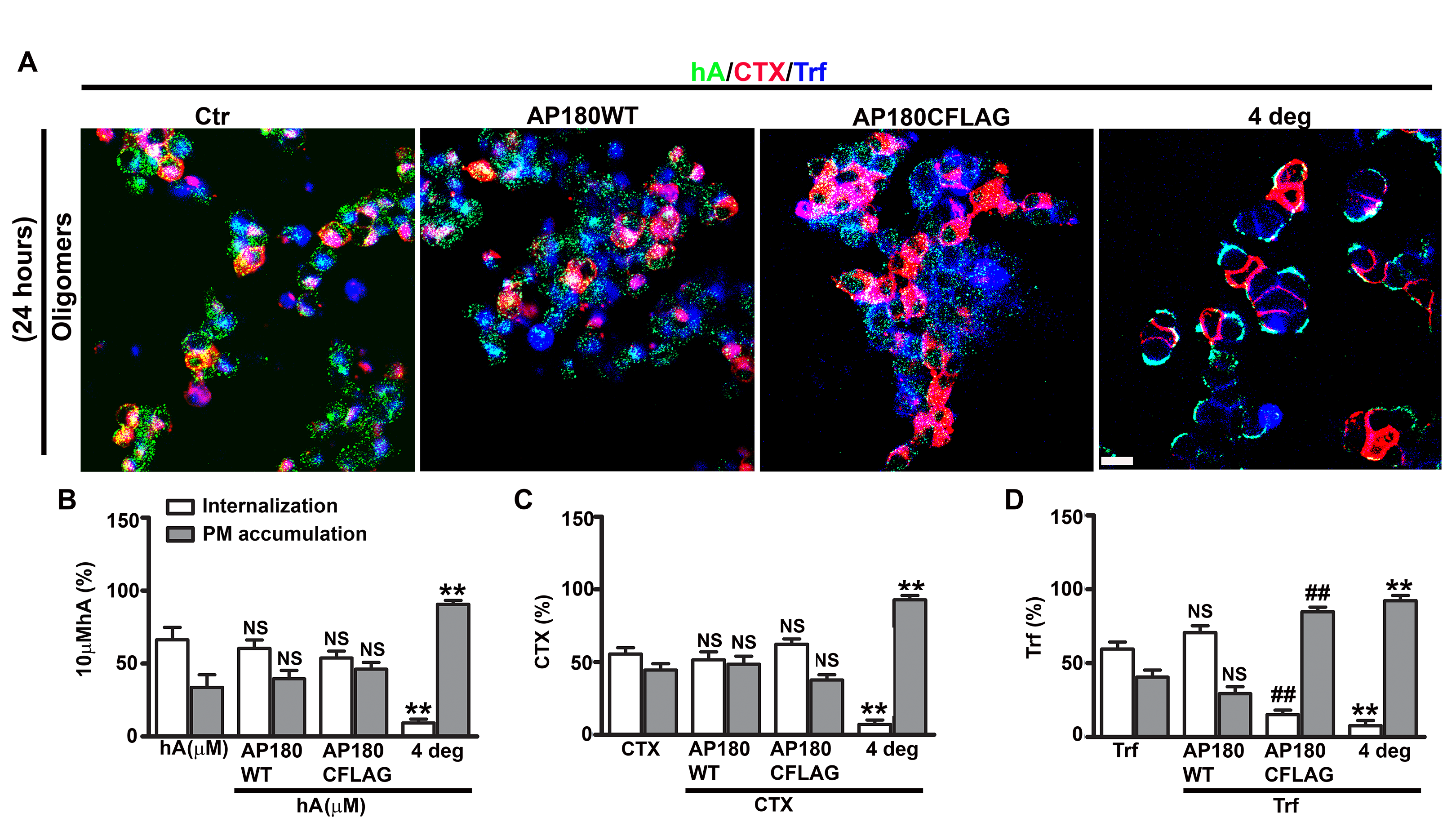

Supplement: Figure S10 — Late entry of amylin oligomers is independent of clathrin in RIN-m5F cells. Cells were transfected with 1µg of wild type (wt-AP180) or DN clathrin adaptor AP180 protein, for 16–18 hours. Following this, cells were incubated with 10 µM human amylin (green) for an additional 24 hours at 37°C. In parallel, human amylin was incubated with cells for 24 hours at 4°C. CTX (red) and Trf (blue) were finally added for additional 30 minutes at 37°C or 4°C. Confocal microscopy (A) and whole cell analysis (B–D) revealed no significant change in cellular distributions of amylin oligomers (B) when transfected with wt-AP180 or DN AP180CFLAG respective to the controls (non-transfected cells). However, there was almost a complete block in internalization of these cytotoxic forms at 4°C. NS P>0.1, hA vs. hA/wt-AP180, NS P>0.1, hA vs. hA/AP180CFLAG and **P<0.01, hA vs. hA/4°C, n = 9. CTX internalization (C) was unaffected with DN AP180CFLAG expression as compared to a marked inhibition in internalization of Trf (D). Both CTX (C) and Trf (D) internalization were further blocked at 4°C, causing an accumulation of these particles on the PM. NS P>0.1, CTX vs. CTX/wt-AP180, NS P>0.1, CTX vs. CTX/AP180CFLAG, **P<0.01, CTX vs. CTX/4°C, NS P>0.1, Trf vs. Trf/wt-AP180, ##P<0.01, Trf vs. Trf/AP180CFLAG and **P<0.01, Trf vs. Trf/4°C, n = 9. Significance established by ANOVA followed by Dunnett-Square test. Bar 10µm. (TIF) [file pone.0073080.s010.tif]

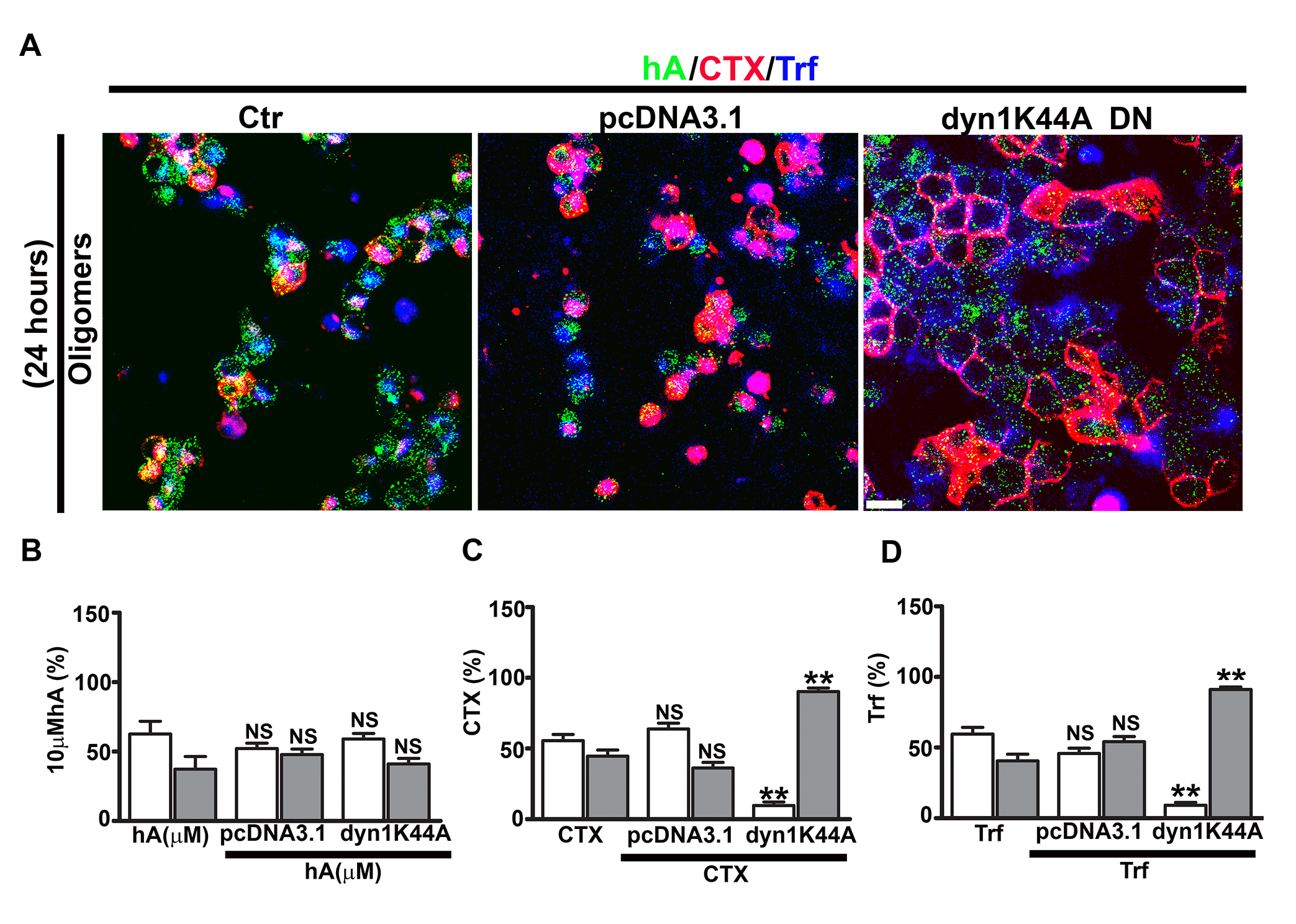

Supplement: Figure S11 — Late phase of amylin oligomer internalization is independent of dynamin in RIN-m5F cells. Cells were transfected with 1µg of the pcDNA3.1 empty vector construct or dynamin mutant, DN dyn1K44Afor 16–18 hours. Cells were then incubated with 10 µM human amylin (green) for additional 24 hours at 37°C. CTX (red) and Trf (blue) were finally added for additional 30 minutes. Confocal microscopy (A) and whole cell analyses (B–D) revealed no significant change in internalization and PM accumulation of amylin oligomers (B) when transfected with either pcDNA3.1 empty vector construct or DN dyn1K44A mutant construct. NS P>0.1, hA vs. hA/pcDNA3.1 and NS P>0.1, hA vs. hA/DN dyn1K44A, n = 9. CTX (C) and Trf (D) were prevented from internalizing these cells when incubated with DN dyn1K44A but not with the pcDNA3.1. NS P>0.1, CTX/Trf vs. CTX, Trf/pcDNA3.1 and **P<0.01, CTX/Trf vs. CTX, Trf/DN dyn1K44A, n = 9. Significance established by ANOVA followed by Dunnett-Square test. Bar 10µm. (TIF) [file pone.0073080.s011.tif]
